# Brassinosteroids regulate root growth by controlling reactive oxygen species homeostasis and dual effect on ethylene synthesis in *Arabidopsis*

**DOI:** 10.1371/journal.pgen.1007144

**Published:** 2018-01-11

**Authors:** Bingsheng Lv, Huiyu Tian, Feng Zhang, Jiajia Liu, Songchong Lu, Mingyi Bai, Chuanyou Li, Zhaojun Ding

**Affiliations:** 1 The Key Laboratory of Plant Cell Engineering and Germplasm Innovation, Ministry of Education, College of Life Science, Shandong University, Jinan, People’s Republic of China; 2 State Key Laboratory of Plant Genomics, National Centre for Plant Gene Research (Beijing), Institute of Genetics and Developmental Biology, Chinese Academy of Sciences, Beijing, People’s Republic of China; Wake Forest University, UNITED STATES

## Abstract

The brassinosteroids (BRs) represent a class of phytohormones, which regulate numerous aspects of growth and development. Here, a *det2-9* mutant defective in BR synthesis was identified from an EMS mutant screening for defects in root length, and was used to investigate the role of BR in root development in *Arabidopsis*. The *det2-9* mutant displays a short-root phenotype, which is result from the reduced cell number in root meristem and decreased cell size in root maturation zone. Ethylene synthesis is highly increased in the *det2-9* mutant compared with the wild type, resulting in the hyper-accumulation of ethylene and the consequent inhibition of root growth. The short-root phenotype of *det2-9* was partially recovered in the *det2-9/acs9* double mutant and *det2-9/ein3/eil1-1* triple mutant which have defects either in ethylene synthesis or ethylene signaling, respectively. Exogenous application of BR showed that BRs either positively or negatively regulate ethylene biosynthesis in a concentration-dependent manner. Different from the BR induced ethylene biosynthesis through stabilizing ACSs stability, we found that the BR signaling transcription factors BES1 and BZR1 directly interacted with the promoters of *ACS7*, *ACS9* and *ACS11* to repress their expression, indicating a native regulation mechanism under physiological levels of BR. In addition, the *det2-9* mutant displayed over accumulated superoxide anions (O_2_^-^) compared with the wild-type control, and the increased O_2_^-^ level was shown to contribute to the inhibition of root growth. The BR-modulated control over the accumulation of O_2_^-^ acted via the peroxidase pathway rather than via the NADPH oxidase pathway. This study reveals an important mechanism by which the hormone cross-regulation between BRs and ethylene or/and ROS is involved in controlling root growth and development in *Arabidopsis*.

## Author summary

Both brassinosteroids (BRs) and ethylene have been known to control root growth and development. ROS have been also reported to play an important role in root development. However, the relationship between BRs and ethylene or ROS in root growth and development was not addressed before. In this study, a *det2-9* mutant defective in BR synthesis was identified from an EMS mutant screening, displaying a short-root phenotype which is result from the hyper-accumulation of ethylene and superoxide anions (O_2_^-^). Exogenous BR apply showed that BRs either positively or negatively regulate ethylene biosynthesis in a concentration-dependent manner. Different from the BR induced ethylene biosynthesis through stabilizing ACSs stability, we found that the BR signaling transcription factors BES1 and BZR1 interacted with promoters of *ACS7*, *ACS9* and *ACS11* to repress their expression, indicating a native regulation mechanism under physiological levels of BR. The BR-modulated control over the accumulation of O_2_^-^ acted via the peroxidase pathway rather than via the NADPH oxidase pathway. This study provides new insights into how brassinosteroids control root growth through the cross-regulation with ethylene synthesis and ROS.

## Introduction

Roots are important plant ground organs, which absorb water and nutrients to control plant growth and development. In higher plants, root growth is maintained by coordinating cell proliferation and differentiation [1–3]. Plant hormones have been known to play a crucial role in the regulation of root growth [4]. Recent studies in the *Arabidopsis* root have shown that different hormones control organ growth by regulating specific growth processes such as cell proliferation, differentiation or expansion in distinct tissues. Plant hormones such as auxin, cytokinin, abscisic acid, brassinosteroids, ethylene and gibberellins have been shown to be involved in root growth through a range of complex interactions. The activities of these hormones during root growth progression depend on cellular context and exhibit either synergistic or antagonistic interactions. For example, ethylene enhances inhibition of root cell elongation through upregulating the expression of *ASA1* and *ASB1* to enhance auxin biosynthesis in *Arabidopsis* seedlings [5]. Furthermore, ethylene regulated root growth was also mediated through modulating the auxin transport machinery [6]. In addition, cytokinin was also found to control root growth through transcriptional regulation of the *PIN* genes and thus influencing auxin distribution [7]. The balance between auxin and cytokinin signaling is crucial during root growth. In *Arabidopsis*, cell division and cell differentiation largely determines root meristem size, which is under the control of cytokinin and auxin through an ARR1/SHY2/PIN circuit [1]. All these studies suggest that hormonal cross-talk plays a pivotal role in the regulation of root growth.

The brassinosteroids (BRs) represent a class of phytohormones involved in a wide variety and developmental processes including root development [8–12]. BR, detected by the BRI1 receptor, activates the transcription factors BES1 and BZR1, which in turn govern the transcription of a large number of genes [13–16]. BRs are known to participate in root growth and development, because mutants impaired with respect to either the synthesis or signaling of BR develop foreshortened roots [17, 18]. However, excessive application of bioactive BR hampered normal development of plants [19]. Therefore, a finely tuned cellular regulation of BR levels is important for the development of plant. It has been found that BR deficient conditions elicit the expression of BR biosynthesis genes, while increase in endogenous BR concentration lead to feedback regulation of the expression of BR metabolic genes to maintain the homeostasis of BR [20]. Recent studies demonstrate that BR interacts with plant hormones such as abscisic acid, gibberellins, auxin and cytokinin to regulate plant growth and development [21–23]. BR interacts with ethylene to regulate the gravitropic response of the shoot, and is involved in ethylene-controlled processes in the hypocotyl of both light- and dark-grown seedlings [24, 25]. Exogenously supplied BR enhances the stability of type 2 of the enzymes 1-aminocyclopropane-1-carboxylate synthase (ACS5 and ACS9) and thus increasing ethylene production, thereby modulating the hypocotyl growth of etiolated seedlings [26]. Though both BRs and ethylene have been reported to regulate root growth and development, it is still unknown if there is a cross-regulation between BRs and ethylene in this process.

In addition to plant hormones, the regulation of root growth has also been tightly linked to reactive oxygen species (ROS). Root growth is profoundly affected by endogenously generated ROS. While ROS were initially believed to merely represent a damaging by-product of the plant’s stress response [27], they have been now recognized as signaling molecules [28]. For example, ROS have been shown to be important for balancing cell proliferation and differentiation during root growth, and have been proposed to adopt a signaling role during lateral root formation [29, 30]. It has been reported that ROS produced in mitochondria of root tip cells in response to the hormone abscisic acid (ABA) are responsible for regulating the root’s meristematic activity [31]. A BR receptor-mediated increase of the cytosolic concentration of calcium ions (Ca^2+^) regulates ROS production, thereby reducing the length of the hypocotyl in dark-grown seedlings [32, 33]. Though BRs have been reported to regulate many plant biotic and abiotic stresses through the regulation of ROS homeostasis [27, 34], the role of the cross-regulation between BRs and ROS in root growth is largely unknown.

Here, the participation of BR in root growth and the extent of its cross-regulation with both ethylene and ROS signaling were investigated by characterizing a novel *A*. *thaliana det2* mutant allele (*det2-9*) selected on the basis of its short-root phenotype, which proved to be defective with respect to BR synthesis. A key observation was that the *det2-9* mutant accumulated more ethylene and ROS than the wild type. The increased accumulations of both ethylene and ROS caused the short root phenotype in *det2-9*. This study reveals a mechanism about how BRs regulate root growth through a cross-regulation with ethylene and ROS signaling.

## Results

### Isolation and characterization of a short-root mutant

**Fig 1:**
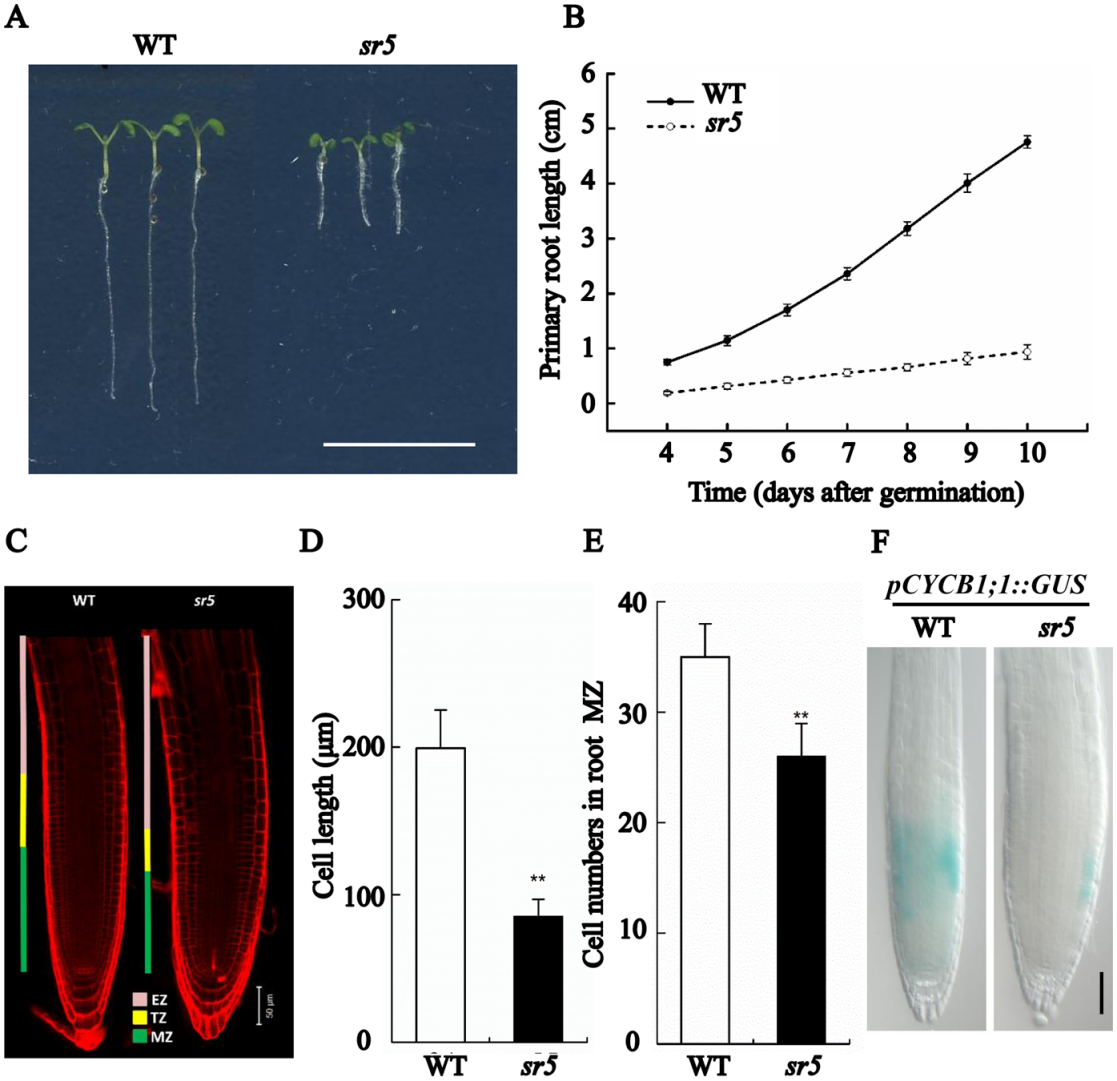
The root of *sr5* mutant growth is restricted and its root meristem size is reduced. (A) The phenotype of five-day old WT and *sr5* seedlings. Bar = 1 cm. (B) Primary root growth over the first ten days following germination. Data shown are mean±SE (n = 25). (C) Longitudinal zonation pattern in the primary roots of five-day old WT and *sr5* seedlings. Cell boundaries appear red following Propidium Iodide staining. The meristem zone (MZ) and transition zone (TZ), which together form the root apical meristem (RAM), and the elongation zone (EZ) are indicated. Bar = 50 μm. (D) Root cortical cell length in the maturation zone of five-day old WT and *sr5* seedlings. Data shown are mean±SE (n = 100), **: means of *sr5* and WT differ significantly (*P*<0.01). (E) Cell number in the proliferation domain of five-day old WT and *sr5* seedlings. Data shown are mean±SE (n = 25), **: means of *sr5* and WT differ significantly (*P*<0.01). (F) *pCYCB1;1*::*GUS* expression in root tips of five-day old WT and *sr5* seedlings. Bar = 50 μm.

To identify novel determinants involved in the control of root growth, an ethyl methane sulfonate (EMS)-mutagenized *Arabidopsis* population was screened by monitoring root length and elongation. One mutant was subsequently named as *short root 5* (*sr5*) (Fig 1A and 1B). The length of the mutant root was only 23% of the one of a wild type (WT) seedling at 7–8 days post germination. A longitudinal zonation pattern analysis showed that the size of its root apical meristem (RAM) was significantly smaller than the WT control (Fig 1C). Both meristem zone (MZ) and transition zone (TZ) in the RAM were substantially reduced in size. Cortical cells in the mutant mature zone were significantly shorter than those in WT, and cell number in the MZ was strongly reduced (Fig 1D and 1E and Table 1). The number of cells formed by the RAM in *sr5* was 1.8 fold fewer than that in the WT control. The length of the mutant’s RAM was 67% of WT’s, and both its MZ and TZ were reduced in size (Table 1). The compromised RAM in the mutant was accompanied by an increased cell cycle time which displayed 1.4 times longer than that in the WT control (Table 1). The signal obtained from the mitotic cyclin B1;1 G_2_/M transition marker *pCYCB1;1*::*GUS* was much weaker in the mutant than the WT control (Fig 1F), an indication that cell proliferation was inhibited in *sr5*. The conclusion was that the mutant’s short root derived from both a reduced MZ cell number and a smaller cell size in the mature zone.

**Table 1:** WT and *sr5* root growth and comparative analysis of their RAM activity.

Genetype	RAM length (μm)	MZ length (μm)	MZ no. of cells	TZ length (μm)	Elongated cell length (μm)	Rate of root growth (μm/h)	Cell production rate (cell h^-1^)	Cell cycle duration (h)
WT	335±33A	209±28A	35±3A	126±17A	199±26A	327.8±48.6A	1.6±0.3A	14.6±3.2a
*sr5*	222±18B	149±7B	26±3B	73±7B	85±11B	74.4±16.8B	0.9±0.2B	20.4±5.9b

Different letters associated with values indicate a significant (upper-case letter: *P*<0.01, lower-case letter: *P*<0.05) difference between the WT and *sr5* means, based on Duncan’s multiple range test.

### The mutated *sr5* gene encodes DET2, a steroid 5α-reductase in the BR synthesis pathway

When positional cloning was employed to identify the site of the *sr5* mutation, a position on chromosome 2 flanked by the markers W20 and W22 was identified (S1A Fig). Sequencing of the genes present in the critical genomic region revealed that the mutant had a point mutation causing a G-to-A transition at nucleotide position 107 after ATG in *At2g38050* (*DET2*). The root growth and seedling morphology of the reported *det2-1* mutant were indistinguishable from those of *sr5* (S1B and S1E Fig). Since the F_1_ hybrid *sr5* x *det2-1* retained the short-root phenotype (S1C Fig), it was concluded that the *sr5* mutation likely involved a lesion in *DET2*. Moreover the *DET2* promoter driving *DET2* cDNA fused to GFP-GUS (*pDET2*::*DET2-GFP-GUS*) complemented the short-root phenotype in *sr5* (S1D Fig), suggesting that the G107A mutation in *DET2* led to the short-root phenotype in the *sr5* mutant. In seedlings carrying the transgene, GUS activity was detected both in the shoot and the RAM (S2 Fig). *DET2* encodes a steroid 5α-reductase acting in the BR synthesis pathway. The phenotype of *sr5*, which was similar to that observed in *det2-1* grown in darkness, was rescuable when the plants were treated with exogenous BR (eBL) (S1B Fig). Since there are eight alleles of *det2* mutant have been reported, we renamed the *sr5* mutant as the *det2-9* which was used for most of the analysis in this study. We compared the expression levels of some BR induced genes between *det2-9* and *det2-1* through Q-PCR analysis. The results showed that, though both mutants displayed reduced expression levels of *TCH4*, *BAS1*, *IAA17* and *IAA19*, the *det2-9* mutant has a higher expression level of these BR-induced genes than the *det2-1* mutant (S3 Fig). Consistently, the *det2-9* mutant had a weaker phenotype compared with the *det2-1* mutant (S1 Fig), indicating that the point mutation at position 107 might not be a null allele.

### Both ROS-responsive and ethylene-related genes were affected in *det2-9*

**Fig 2:**
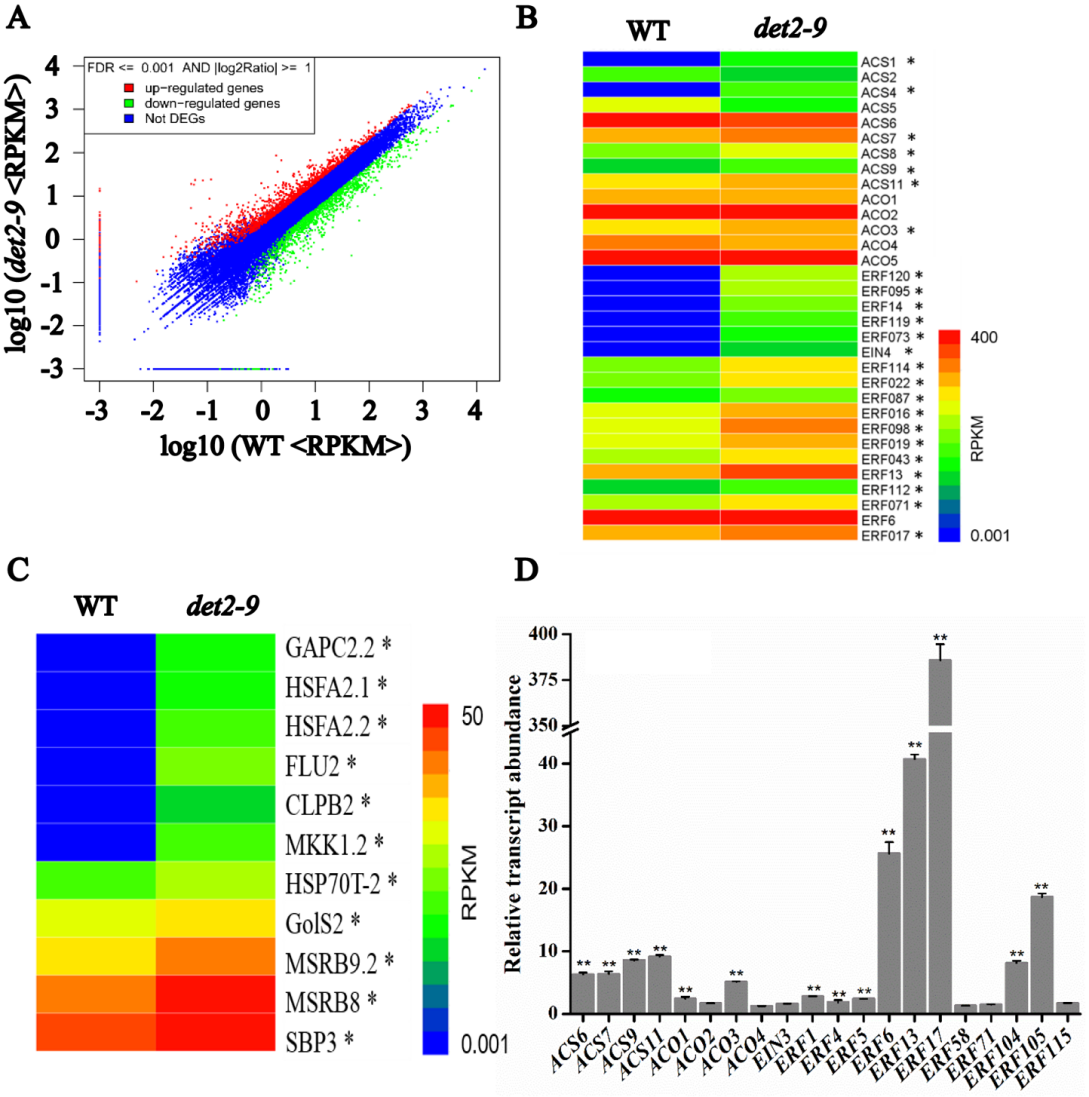
RNA-Seq analysis of the *det2-9* and WT root transcriptome. (A) Differential transcription in *det2-9* and WT. Genes, which were significantly induced or repressed in *det2-9* compared with WT, appear in, respectively, the upper left (in red) and bottom right (in green) hand portions of the plot. RPKM: reads per kilobase per million reads, DEGs: differentially expressed genes. (B, C) A heat map representation of the transcriptional behavior of genes associated with ethylene (B) and responding to ROS (C) based on the GO analysis. *: means more than two times up change in the expression genes of *det2-9* compared to WT. (D) A qRT-PCR expression analysis of a selection of genes related to ethylene in *det2-9* compared to WT. Data shown are mean±SE (n = 3), **: means of *det2-9* and WT differ significantly (*P*<0.01).

A RNA-seq approach was applied to compare the *det2-9* root transcriptome with that of the WT, a total of 1,480 and 1,116 genes were found to be, respectively, up- and down-regulated (Fig 2A). Among the differentially expressed genes, based on the GO analysis we found that there is a statistically significant enrichment in genes annotated as being linked to secondary metabolic process and response to stimulus (*P*<0.01). It is not surprising for this enrichment considering the dwarf phenotype of the mutant. Though the previous research has shown that exogenously supplied BR can enhance the production of ethylene [26], the ethylene biosynthesis and ethylene response factors were up-regulated in *det2-9* according to our RNA-seq analysis (Fig 2B). These genes included 1-aminocyclopropane-1-carboxylate synthase encoding genes, 1-aminocyclopropane-1-carboxylate oxidase encoding genes and ethylene response factor encoding genes (S1 Dataset). According to our GO analysis, we also found that many of genes belong to GO:0000302 (response to reactive oxygen species) were up-regulated significantly in *det2-9* (Fig 2C), which was in contrast with the previous reports showing that BR could induce the generation of H_2_O_2_ [27, 34]. To confirm the RNA-seq results, we performed a quantitative real time-PCR (qRT-PCR) assay on a selection of 20 ethylene related genes which were differentially transcribed in WT and *det2-9* seedlings grown in light and dark growth conditions (Fig 2D and S4 Fig). Though the expression changes of most of ethylene related genes were confirmed, we also found that some genes, for example *ACS6*, *ERF6* and *ERF17*, had little agreement between the transcript abundance by RNA-seq and qRT-PCR analysis. Considering three independent repeats were done for the confirmations, the results of qRT-PCR analysis are more reliable. These results suggest that both ROS and ethylene signaling were enhanced in the *det2-9* mutant.

### BRs either positively or negatively regulate ethylene biosynthesis in a concentration-dependent manner

**Fig 3:**
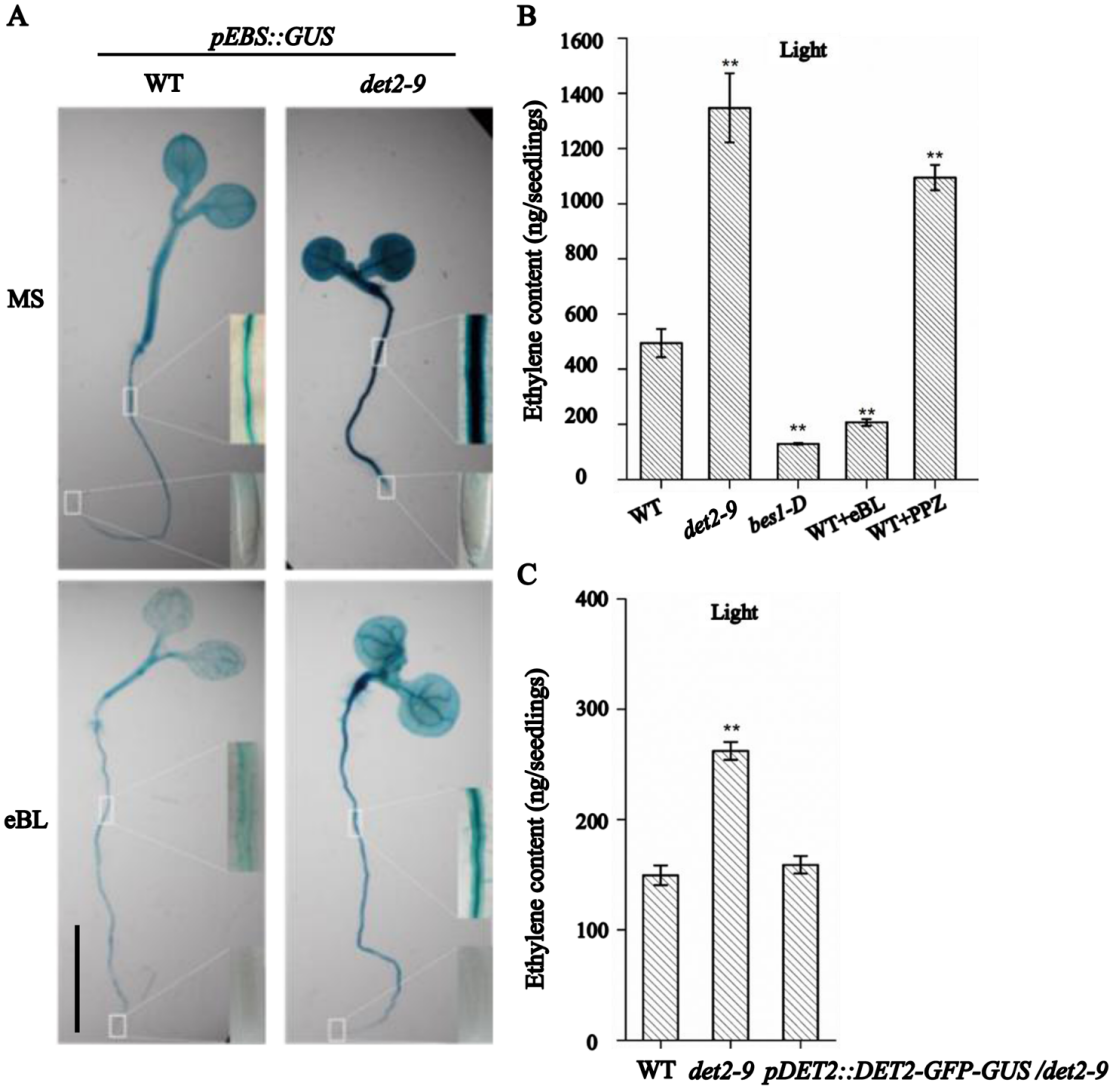
The *det2-9* mutant accumulates more ethylene than WT. (A) Ethylene-induced GUS activity (*pEBS*::*GUS*) in *det2-9* and WT. Seedlings of *det2-9* and WT were grown for five days either in the presence or absence of 10 nM eBL, and were stained for GUS activity analysis. Each treatment involved 20–30 seedlings; here, representative samples are presented. Bar = 1 cm. (B) Ethylene content in indicated BR-related transgenic and WT nine-day old seedlings exposed to either eBL (10 nM) or propiconazole (2 μM) under light conditions. Data shown are mean±SE (n = 5). **: means of *det2-9*, *bes1-D*, WT+eBL, WT+PPZ differ significantly from mean of WT (*P*<0.01). (C) Ethylene content in five-day old WT, *det2-9* and *pDET2*::*DET2-GFP-GUS*/*det2-9* seedlings in light conditions. Data shown are mean±SE (n = 5). **: means of *det2-9* and WT differ significantly (*P*<0.01).

Ethylene signaling in the *det2-9* mutant was monitored by the expression of the *pEBS*::*GUS* ethylene signaling reporter. The strength of the GUS signal was considerably higher in the mutant than that in the WT (Fig 3A), suggesting that an enhanced level of ethylene signaling occurred in *det2-9*. The increased ethylene response in *det2-9* was abolished by the presence of 10 nM eBL during seedling growth (Fig 3A). The ethylene content was considerably higher in the *det2-9* mutant than that in WT seedlings (Fig 3B and 3C). The transgene line *pDET2*::*DET2-GFP-GUS/det2-9* complemented the higher level of ethylene observed in *det2-9* (Fig 3C). Treatment with the BR synthesis inhibitor propiconazole (PPZ) also resulted in higher ethylene content in light-grown WT seedlings, while eBL (10 nM, a concentration which partially rescued the short-root phenotype in *det2-9*) treated WT or *bes1-D* (a mutant which displays an enhanced BR signaling response) light-grown seedlings both showed a reduction in ethylene content (Fig 3B). A similar profile of ethylene content was also observed when seedlings were grown in darkness (S5 Fig). All these above chemical treatment experiments and mutant analysis suggest that both exogenous applied low levels of BR and native BR signaling negatively regulated ethylene biosynthesis.

**Fig 4:**
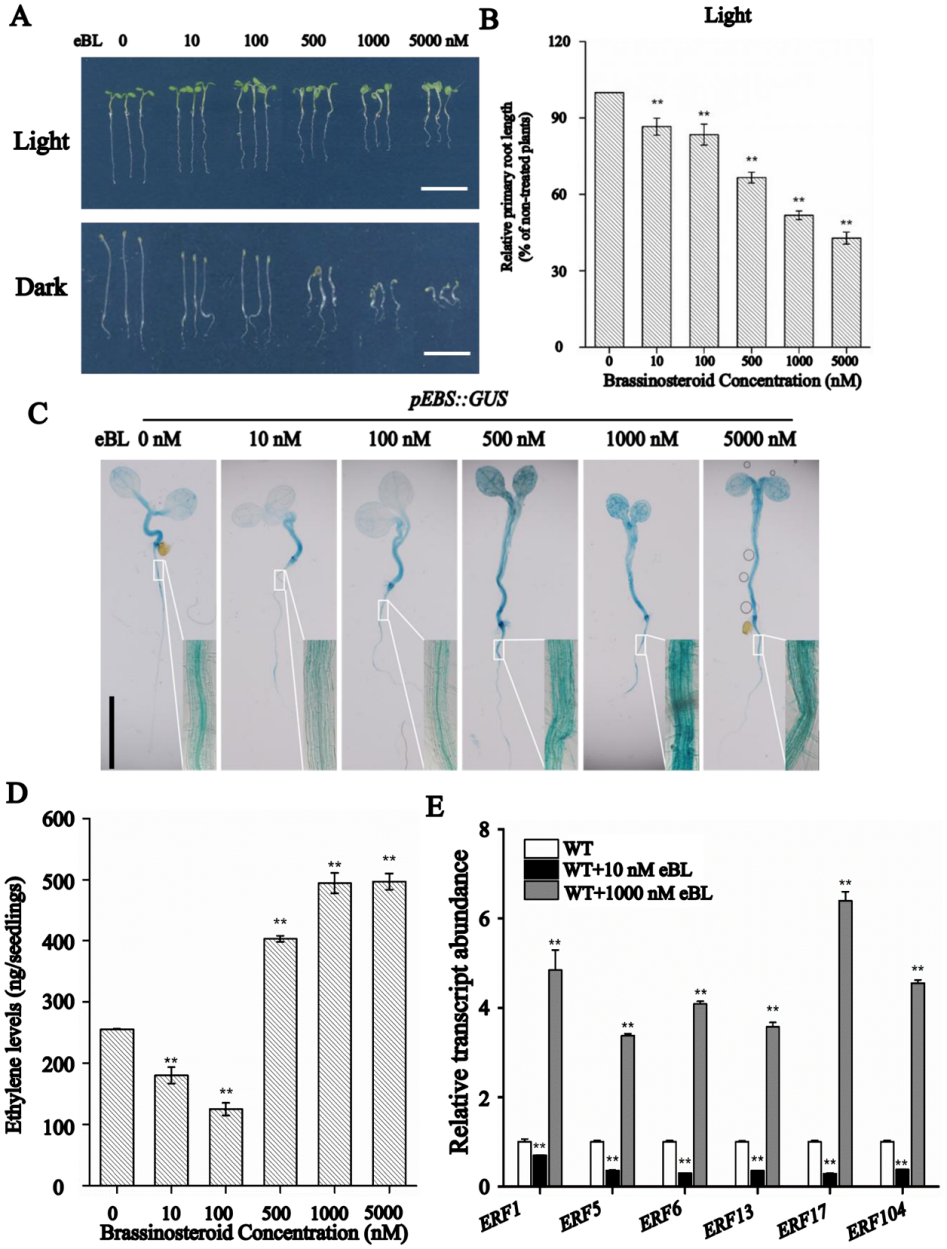
BR affects root growth and ethylene production in *Arabidopsis*. (A) and (B) Phenotype of five-day old Col-0 seedlings grown in different concentrations of BR. Bar = 1 cm. Data shown are mean±SE (n = 30). **: means significantly different in treated seedlings versus control (*P*<0.01). (C) Ethylene-induced GUS activity (*pEBS*::*GUS*) in seedlings grown on different concentration of eBL. Seedlings of *pEBS*::*GUS* were grown for five days in the presence of different concentrations of eBL, after which they were stained for GUS activity. Each treatment involved 20–30 seedlings; here, representative samples are presented. Bar = 1 cm. (D) Ethylene levels in seedlings grown in the presence of different concentration of eBL. Data shown are mean±SE (n = 30). **: means in treated seedlings significantly differ from control (*P*<0.01). (E) Transcript abundance of *ERF*s in WT grown in different concentration of eBL. **: means in treated seedling significantly differ from untreated samples (*P*<0.01).

In addition, we also observed that root growth was inhibited gradually by eBL at concentrations ranging from 10 to 5000 nM (Fig 4A and 4B). While the hypocotyl length was unchanged when treated with low concentration of eBL (<500 nM) but reduced sharply when the concentration of eBL greater than 500 nM (Fig 4A). Furthermore, dark-grown seedling hypocotyls treated with higher concentration of eBL (≥500 nM) displayed a typical “triple response”, indicating the enhanced ethylene response (Fig 4A). Therefore, we further examined the effects of BR on ethylene production using different concentrations of eBL. The results showed that ethylene content was greatly reduced in seedlings treated with low concentration of eBL (10 or 100 nM) while it was strongly increased when the concentration of eBL greater than 500 nM (Fig 4D). Consistently, both GUS staining analysis with the *pEBS*::*GUS* transgene line and an examination of ethylene response factors (ERFs) expression using qRT-PCR analysis show that low concentrations (10–100 nM) of BR inhibits ERF expression while high concentrations (≥500 nM) of BR enhanced expression (Fig 4C and 4E), consistent with the change ethylene levels. In summary, BR either positively or negatively regulate ethylene biosynthesis depends on the levels of BRs.

### The enhanced ethylene response contributes to the short-root phenotype in *det2-9*

**Fig 5:**
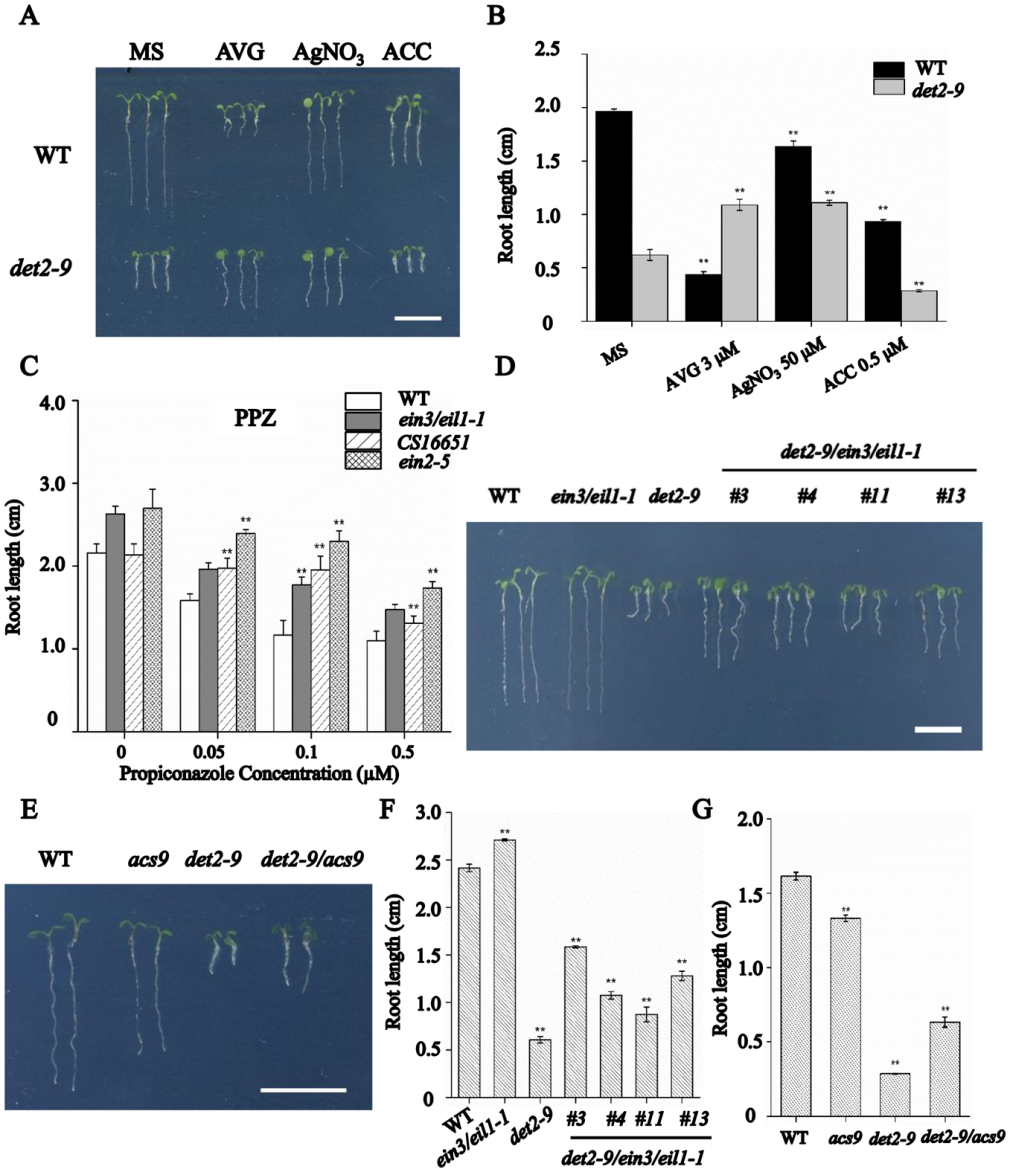
Enhanced ethylene synthesis is involved in the inhibitory effect of BR on root growth. (A, B) Root growth of WT and *det2-9* in the presence of either AVG, AgNO_3_ or ACC under light conditions. Data shown in B are mean±SE (n = 30); **: means of *det2-9* and WT differ significantly (*P*<0.01). Bar = 1 cm. (C) Root growth in the presence of propiconazole (2 μM) of the octuple *acs* mutant *CS16651*, the *ein2-5* mutant and the *ein3/eil1-1* double mutant. Data shown are mean±SE (n = 30); **: means of *CS16651*, *ein2-5*, *ein3/eil1-1* and WT differ significantly (*P*<0.01). The root phenotype (D) and root length measurement (F) of five-day old WT, *ein3/eil1-1*, *det2-9* and four lines of *det2-9/ein3/eil1-1* triple mutant seedlings. Bar = 1 cm. Data shown are mean±SE (n = 30); **: means significantly differ from WT (*P*<0.01). The root phenotype (E) and root length measurement (G) of five-day old WT, *acs9*, *det2-9* and *det2-9/acs9* double mutant seedlings. Bar = 1 cm. Data shown are mean±SE (n = 30); **: means significantly differ from WT (*P*<0.01).

When *det2-9* mutant seedlings were grown on a medium containing either silver nitrate (AgNO_3_, an antagonist of ethylene signaling) or 2-aminoethoxyvinyl glycine (AVG) (an inhibitor of ethylene synthesis), the root growth of *det2-9* mutant was partially rescued, producing root lengths almost double than those developed by the non-treated mutant seedlings. However, both treatments inhibited the root growth of WT seedlings (Fig 5A and 5B). In addition inhibition of ethylene signaling by AgNO_3_ rescued the cortical cell length in *det2-9* (S6 Fig). On the other hand, the root cell elongation and root growth of the mutant seedlings was found to be more sensitive to ACC (a precursor of ethylene synthesis) (Fig 5A and 5B and S6A Fig). In addition, both WT and *det2-9* mutant seedlings displayed similar cell numbers in root meristem under the same treatment with either AgNO_3_ or ACC (S6B Fig). This result suggests that the BR deficiency caused short-root phenotypes in *det2-9* was mediated by the effect of ethylene signaling on root cell elongation. Consistently, the octuple *acs* mutant *CS16651* (*acs2-1/acs4-1/acs5-2/acs6-1/acs7-1/acs9-1/amiRacs8acs11*), *ein2-5* and the *ein3/eil1-1* double mutant, which have defects in either ethylene biosynthesis or ethylene signaling, were less affected by the PPZ treatment than WT (Fig 5C). The short-root phenotype of *det2-9* was partially recovered in the *det2-9/acs9* double mutant and *det2-9/ein3/eil1-1* triple mutant (Fig 5D–5G). These results indicate that the short-root phenotype in *det2-9* partly result from enhanced ethylene biosynthesis and ethylene signaling.

### BR regulates ethylene biosynthesis via BZR1/BES1-mediated repression of *ACS*

**Fig 6:**
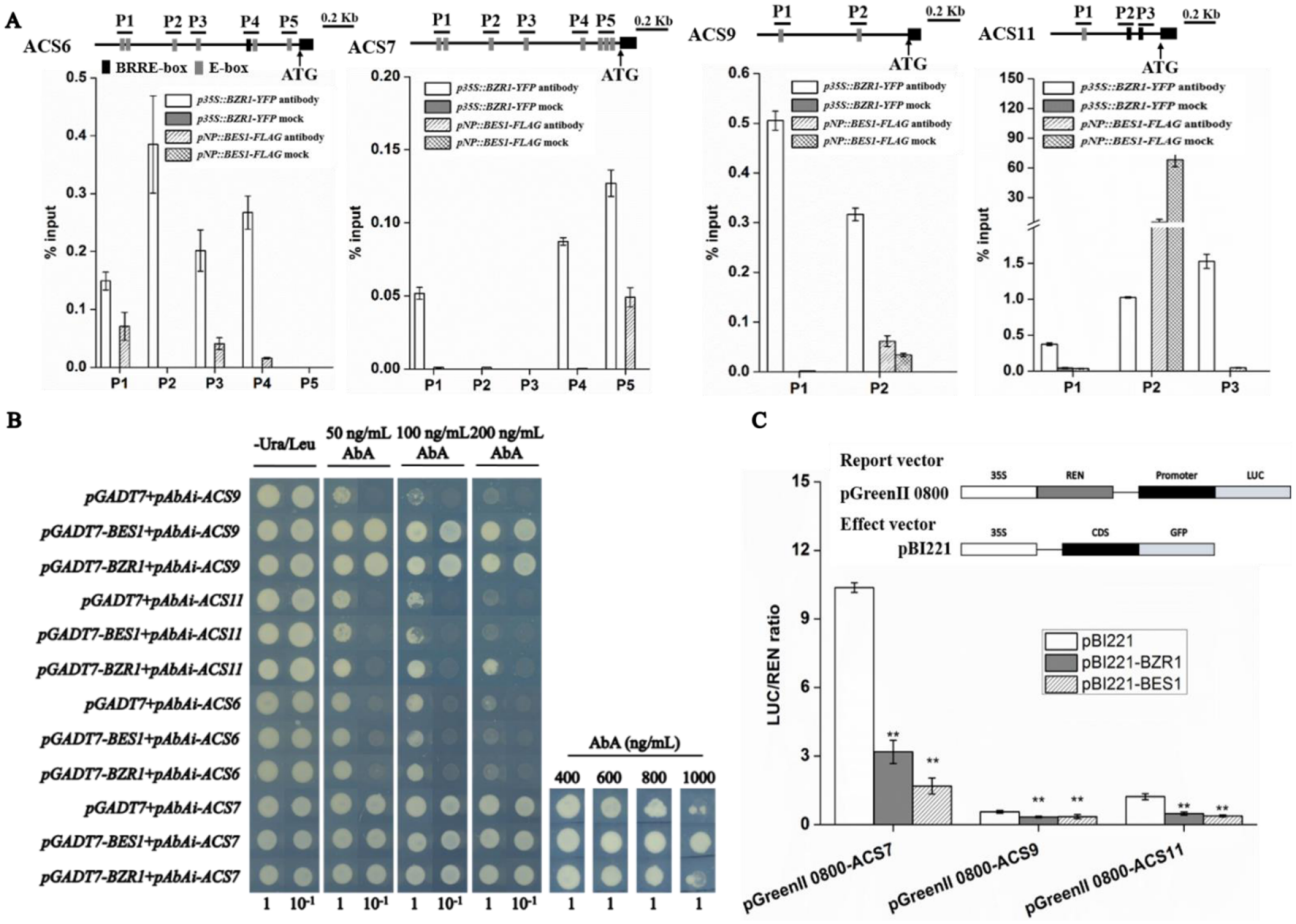
Interaction of BZR1/BES1 transcription factors with various *ACS* promoters. (A) ChIP/qPCR assay. A scheme of the promoters of *ACS6*, *7*, *9* and *11* are shown with the position of BRRE-box (black) and E-box (gray). Graphs show the ratio of bound promoter fragments (P1-P5) versus total input detected by qPCR after immuno-precipitation in *p35S*::*BZR1-YFP* and *pNP*::*BES1-FLAG* seedlings by YFP or FLAG antibodies. Data shown are mean±SE (n = 9). (B) Yeast one-hybrid binding assay involving BZR1/BES1 and *ACS6*, *7*, *9* and *11* promoters. (C) Transient expression in *A*. *thaliana* protoplasts. BZR1 or BES1 transcription factors were co-transfected with either *ACS7*, *9* or *11* promoters. The LUC to REN ratio is shown and indicated the activity of the transcription factors on the expression level of the promoters. LUC: firefly luciferase activity, REN: renilla luciferase activity. Data shown are mean±SE (n = 9); **: means significant difference compared to control (*P*<0.01).

A promoter analysis showed that promoters of *ACS6*, *7*, *9*, *11*, along with *ACO1* and *3* (all these genes were strongly up-regulated in *det2-9*, Fig 2) contained a BRRE and/or an E-box, the binding sites for BES1 and BZR1 (Fig 6A). The direct interaction of the *ACS*s by BES1 or BZR1 was confirmed by a chromatin immunoprecipitation (ChIP)/qPCR analysis in FLAG-tagged BES1 or YFP-tagged BZR1 transgenic lines (Fig 6A). A series of yeast one-hybrid assays were conducted to further verify whether any of these promoters was regulated directly by either BES1 or BZR1. The outcome was that in yeast BES1 interacted with the promoters of *ACS7* and *9*, while BZR1 did so with the promoters of *ACS9* and *11* (Fig 6B). Neither of the two transcription factors interacted definitively with the *ACS6* (Fig 6B), *ACO1* or the *ACO3* promoter (S7 Fig). The trans-activity of BES1 or BZR1 with the *ACS* promoters was further demonstrated in a transient dual LUC expression assay in *A*. *thaliana* mesophyll protoplasts. The over-expression of both BES1 and BZR1 strongly repressed the activity of *ACS* promoters (Fig 6C), confirming that either BES1 or BZR1 can repress *ACS7*, *9* and *11* gene expression *in vivo*.

A previous study reported that short-term treatment with eBL resulted in dephosphorylation of BES1 (its active form) in WT and in *det2-1*, but not in *bri1-5 or bin2-1* signaling BR mutants [35]. Therefore we further investigate the expression of *ACS*s in BR signaling mutants including *bri1-116* and *bin2-1*. qRT-PCR results showed that the expression of *ACS6*, *7*, *9* and *11* increased in the *det2-9* (see also Fig 2D), *bri1-116* and *bin2-1* mutant compared with WT (S8 Fig). The expressions of these four *ACS* genes decreased when treated with eBL to a lower extent in *bri1-116* or *bin2-1* mutants compared with the *det2-9* mutant (S8 Fig). These results indicate that BR signaling pathway is required for the BR-mediated repression of *ACS* gene expression, via direct regulation by the BES1 and BZR1 transcription factors.

### The short-root phenotype in *det2-9* was partly attributed to the hyper-accumulation of O_2_^-^

**Fig 7:**
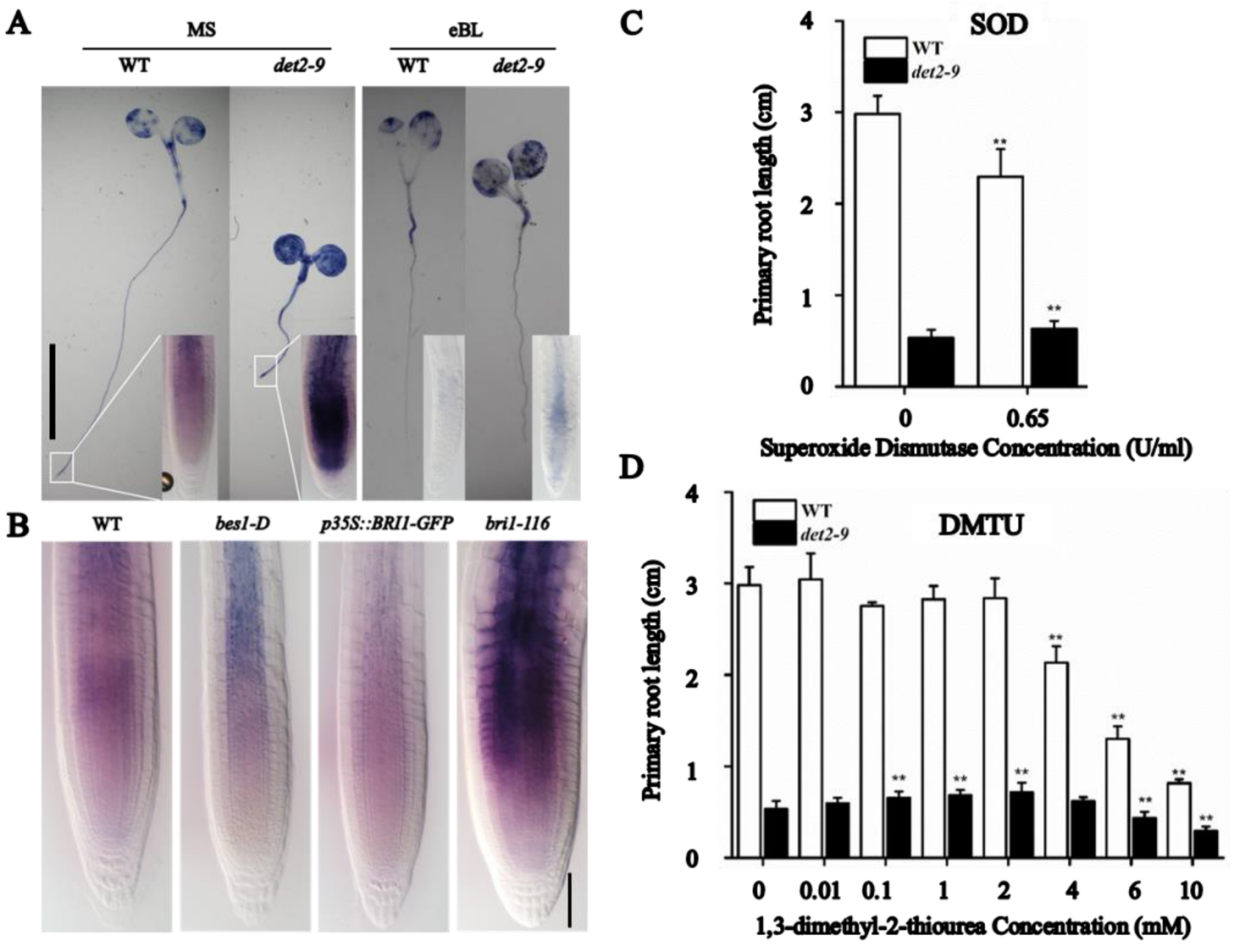
BR represses the accumulation of superoxide anions. (A) Five-day old *det2-9* and WT seedlings grown in the presence or absence of eBL (10 nM), then assayed for the superoxide anion using NBT. Bar = 1 cm. (B) Superoxide anion accumulation in the root tips of BR-signaling enhanced plants (*p35S*::*BRI1-GFP* and *bes1-D*) and BR signaling deficient plants (*bri1-116*). Bar = 50 μm. (C, D) Elongation of the primary root of WT and *det2-9* seedlings exposed to either (C) superoxide dismutase (SOD) or (D) 1,3-dimethyl-2-thiourea (DMTU). Data shown are mean±SE (n = 30); Asterisks means significant difference from the control-treated plants (**P<0.01).

The transcriptomic analysis in *det2-9* mutant roots identified genes responding to ROS as BR-targets (Fig 2C). Therefore, we further analyzed *det2-9* mutant for defects in ROS using the nitroblue tetrazolium (NBT) staining method to detect the presence of O_2_^-^ *in vivo* [36]. The NBT signal was higher in the *det2-9* mutant than that in the WT control (Fig 7A), while there was no clear difference when 3,3’-diaminobenzidine (DAB) staining was used to visualize the level of H_2_O_2_ present [37] (S9 Fig). This suggests that *det2-9* accumulated O_2_^-^ but not H_2_O_2_. Treatment with eBL substantially reduced the extent of the O_2_^-^ hyper-accumulation in *det2-9* (Fig 7A). Meanwhile, the BR-signaling defective mutant *bri1-116* hyper-accumulated O_2_^-^, while BR-signaling enhanced plants (*p35S*::*BRI1-GFP* or *bes1-D*) accumulated less superoxide anion in their roots compared with WT plants (Fig 7B), indicating that BR signaling suppresses the accumulation of O_2_^-^. Therefore, root growth analysis was done using *det2-9* mutant seedlings were exposed to two different O_2_^-^ scavengers, namely superoxide dismutase (SOD) [38] and 1,3-dimethyl-2-thiourea (DMTU) [39]. The root length in *det2-9* was significantly increased in the presence of 0.65U/ml SOD, while the same treatment inhibited root growth in WT seedlings (Fig 7C). Similarly, a concentration of 0.1 to 2 mM DMTU treatment, which has no effect on root growth in WT seedlings, could significantly increase root lengths in *det2-9* (Fig 7D).

### The peroxidase pathway, but not the NADPH oxidase pathway, is required for the hyper-accumulation of O_2_^-^ in *det2-9*

**Fig 8:**
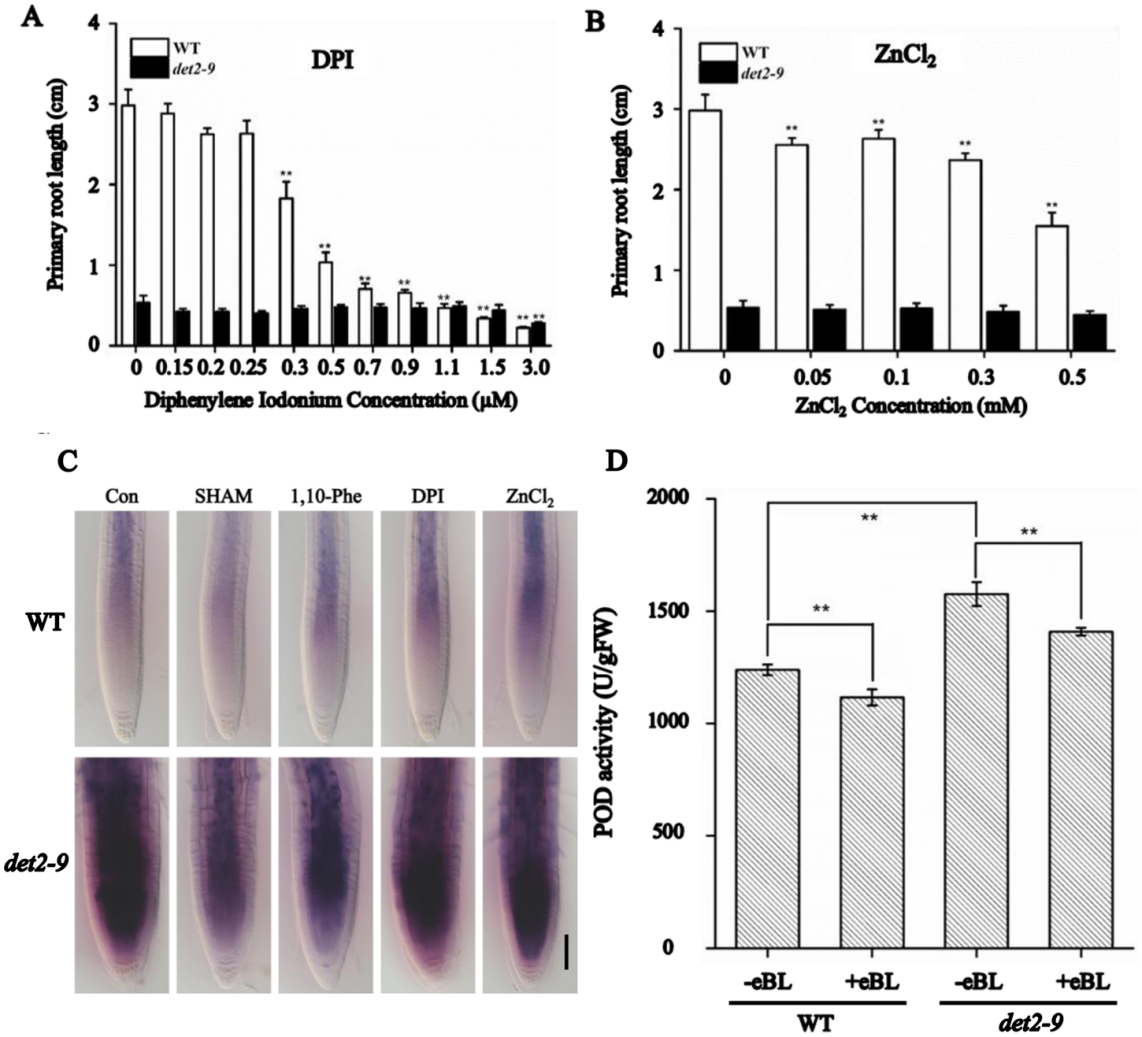
BR inhibits the synthesis of superoxide anions through the peroxidase pathway. The length of the primary root of WT and *det2-9* seedlings when exposed to inhibitors of NADPH oxidase (A) diphenylene iodonium, (B) ZnCl_2_. Asterisks means significant difference from the control-treated plants (**P<0.01). (C) NBT staining of WT and *det2-9* when exposed to salicylhydroxamic acid (SHAM), 1,10-phenanthroline (1,10-Phe), diphenylene iodonium (DPI) and ZnCl_2_. Bar = 50 μm. (D) Peroxidase activity in WT and *det2-9* nine-day old seedlings treated or not with eBL (10 nM). Data shown are mean±SE (n = 6); **: means differ significantly (*P*<0.01).

The accumulation of O_2_^-^ in *det2-9* can be the result of the activation of two signaling pathways: peroxidase or NADPH oxidase. When the NADPH oxidase pathway was blocked by the presence of either diphenylene iodonium (DPI) [40] or ZnCl_2_ [41], *det2-9* mutant roots were insensitive to any treatment (Fig 8A and 8B). Consistent with this result, the abundance of transcripts of the four NADPH oxidase genes (*RBOHC*, *D*, *F* and *G*) was identical in *det2-9* and WT (S10A Fig). The root growth response to PPZ treatment of three mutants *rbohD*, *rbohF* and *rbohD/F* was also similar to the one of WT (S10B Fig). NBT staining showed that the BR deficiency-induced O_2_^-^ hyper-accumulation by PPZ treatment was unaffected in both plants harboring *p35S*::*NADPHD-GFP* and the *rbohD/F* double mutant (S10C Fig). And O_2_^-^ hyper-accumulates in *det2-9/rbohD* and *det2-9/rbohD/F* mutants similarly to *det2-9*, compared with WT (S10D Fig). These experiments allow us to conclude that the hyper-accumulation of O_2_^-^ in *det2-9* did not involve the NADPH oxidase pathway. So attention was focused on the peroxidase pathway [29], by treating seedlings with either salicylhydroxamic acid (SHAM) [42] or 1,10-phenanthroline (1,10-Phe) [43], inhibitors of peroxidase activity. The root length of *det2-9* was significantly increased by both treatments, whereas root growth of WT was slightly inhibited (S11A and S11B Fig). NBT staining showed that the levels of O_2_^-^ in *det2-9* reduced sharply when treated with SHAM or 1,10-Phe but no obvious changes were observed when treated with DPI or ZnCl_2_ (Fig 8C), which was consistent with the NBT staining observed in *det2-9/rbohD* and *det2-9/rbohD/F* mutants compared with WT and *det2-9* (S10D Fig). When the transcription of genes encoding peroxidase was investigated, no clear-cut differences were visible between the mutant and WT (S12 Fig), but peroxidase activity was much stronger in the *det2-9* mutant and was reduced when seedlings were treated with exogenous BR (Fig 8D). Thus the hyper-accumulation of O_2_^-^ in *det2-9* was likely the effects of an increased peroxidase activity.

### Relationship between ethylene and O_2_^-^

**Fig 9:**
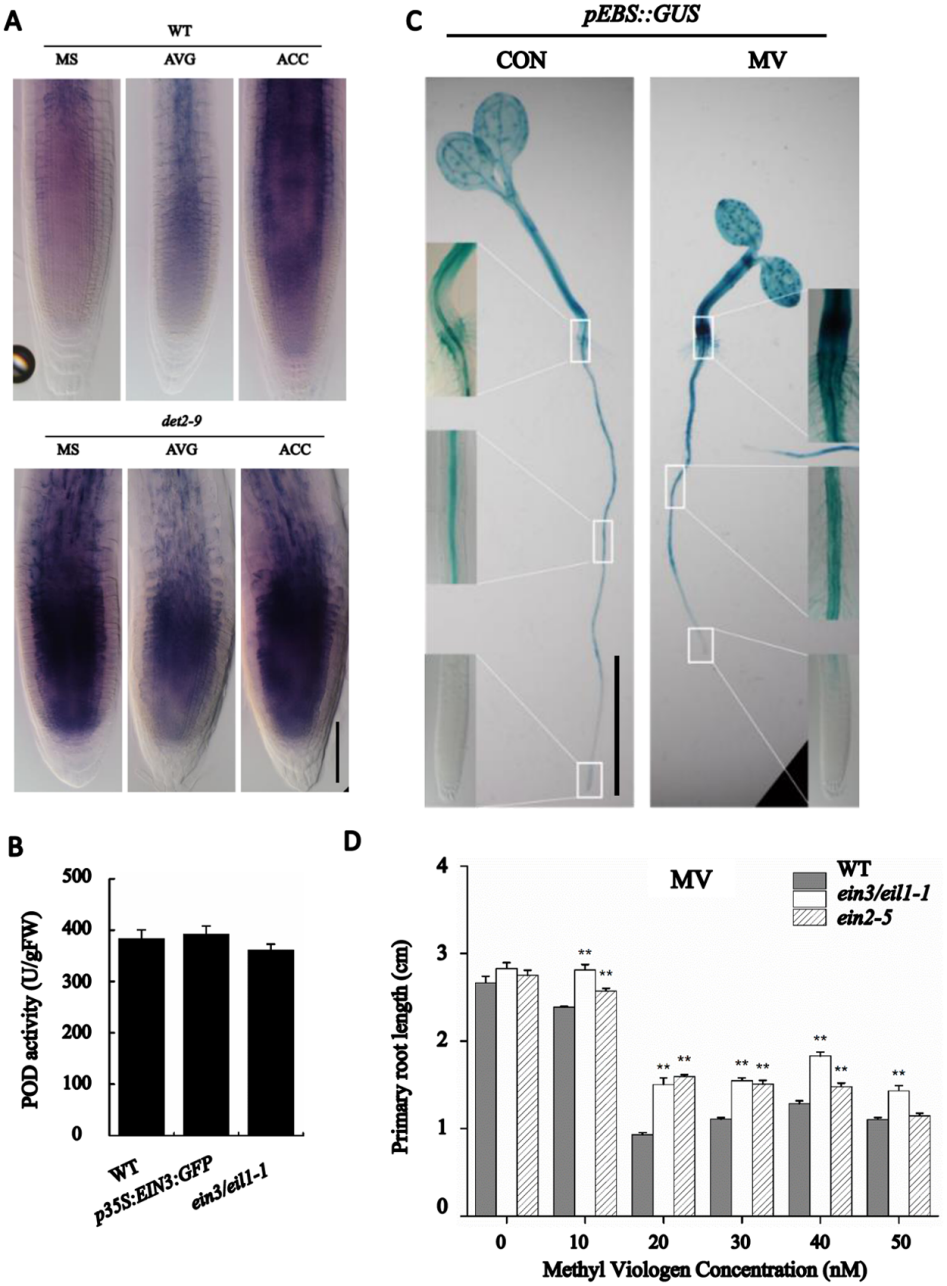
Relationship between ethylene and superoxide anion. (A) NBT staining of WT and *det2-9* seedlings exposed to either AVG or ACC. Bar = 50 μm. (B) Peroxidase activity (POD) in WT, *p35S*::*EIN3-GFP* and the *ein3/eil1-1* double mutant seedlings. Data shown are mean±SE (n = 6); no significant differences calculated. (C) Ethylene-induced GUS activity (*pEBS*::*GUS*) in the WT when treated or not with methyl viologen (MV). Bar = 1 cm. (D) Primary root length of WT, *ein2-5* and *ein3/eil1-1* when treated with various MV concentrations. Data shown are mean±SE (n = 30); **: means significant difference compared to WT (*P*<0.01).

Given that the level of both ethylene and O_2_^-^ was enhanced in *det2-9*, the question arose as to whether ethylene and ROS interacted with one another. O_2_^-^ accumulation was initially assayed in WT and *det2-9* plants treated with either AVG or ACC (Fig 9A). NBT staining showed that the ACC treatment had a positive and AVG had a negative effect on superoxide anion accumulation in WT roots (Fig 9A). This indicates that ethylene induces an accumulation of O_2_^-^ in *Arabidopsis*. NBT staining also showed that *p35S*::*EIN3-GFP* accumulated more O_2_^-^ than WT, but when treated with PPZ, the extent of O_2_^-^ accumulation was similar among *p35S*::*EIN3-GFP*, *ein3/eil1-1* and WT (S13 Fig), indicating that there was another pathway independent from ethylene participating in O_2_^-^ accumulation when BR synthesis was blocked with PPZ treatment. In the *det2-9* mutant, there was no clear increasement for ACC-induced superoxide anion accumulation, but the AVG treatment reduced it, which is also an indication that the increase in superoxide anion accumulation was at least partially dependent on ethylene production in *det2-9*. Since peroxidase activity in *det2-9* was higher than that in WT (Fig 8D), an experiment was conducted to compare peroxidase activity in plants carrying *p35S*::*EIN3-GFP*, the *ein3/eil1-1* double mutant and WT. The result showed that the peroxidase activity was not clearly affected in *p35S*:*EIN3*:*GFP*, *ein3/eil1-1* compared with the wild-type control (Fig 9B), indicating that ethylene signaling pathway is unlikely to activate the POD pathway for BR-regulated accumulation of O_2_^-^ in *det2-9* mutant.

To investigate whether the O_2_^-^ accumulation can alter normal ethylene signaling, we compared the expression level of *pEBS*::*GUS* when treated or not with methyl viologen (MV, a superoxide anion propagator) treatment. As shown in Fig 9C, the expression level of *pEBS*::*GUS* reporter was considerably induced when treated with MV, suggesting that an O_2_^-^ accumulation can increase ethylene content. We then measured the primary root growth of *ein2-5*, *ein3/eil1-1* and wild type when treated or not with MV. Mutants in ethylene signaling were more resistant than WT to the negative effects of MV on root growth (Fig 9D). The expression of genes encoding ACS and ACO, analyzed by qRT-PCR, increased when treated with MV (S14 Fig). These results further indicated that O_2_^-^ accumulation can alter normal ethylene production.

## Discussion

The BRs are well recognized as promoters of cell elongation, also in addition to their involvement in the de-etiolation response, where the opening of the apical hook is thought to require a decrease in the level of ethylene synthesis [17, 44, 45]. It was found that BR enhances ethylene production through the synergistic interaction with *eto1* and *eto3* [46]. Another study from the same lab showed that supplying BR exogenously promotes ethylene synthesis in the *A*. *thaliana* seedlings via stabilizing ACS5 and ACS9 protein [26]. This BR-induced ethylene production was also observed in mung bean and maize [47, 48]. However, in jujube fruit, 5 μM BR-treated fruits caused a significantly lower level of ethylene during storage and the inhibition fruit ripening [49]. These contradictory results indicate the complicated effects of BR on ethylene synthesis. However, all these observations are based on the chemical treatment with BR.

In this study, a new mutant allele of DET2, *det2-9*, was identified based on the short-root phenotype (Fig 1 and S1 Fig). *DET2* encodes a steroid 5α-reductase involved in BR biosynthesis, catalyzing the formation of campestanol with campesterol as substrates. Since another allele *det2-1* and other mutants such as *cpd* and *dwf4* which have defects in different steps of BR biosynthesis also displayed short-root phenotype [50–52], it is unlikely that campesterol accumulation caused the short-root phenotype. The short-root phenotype is most likely a result of the reduced BRs synthesis in *det2-9* since externally applied BRs could largely rescued the mutant phenotypes (S1 Fig). Through genetic analysis and chemical treatment, we found that the short-root phenotype in *det2-9* was partly the resulted of over accumulation of ethylene leading to enhanced ethylene signaling (Figs 3 and 5). The ethylene content was considerably higher in the *det2-9* mutant than that in WT seedlings (Fig 3B). Treatment with the BR synthesis inhibitor propiconazole (PPZ) also resulted in higher ethylene content in light-grown WT seedlings, while eBL (10 nM, a concentration which enhances the growth of root in *det2-9*) treated WT or *bes1-D* (a mutant which displays an enhanced BR signaling response) light-grown seedlings both showed a reduction in ethylene content (Fig 3B). A similar profile of ethylene content was also observed when seedlings were grown in darkness (S5 Fig). Transcriptional profiling showed that a number of *ACS* genes were up-regulated in the *det2-9* mutant both in light and dark growth conditions, consistent with its increased level of ethylene (Figs 2B, 2D and 3 and S4 and S5 Figs). Since the BR signaling transcription factors BZR1 or BES1 bind to *ACS* promoters to repress their expression (Fig 6), we analyzed BR signaling pathway by using *bri1-116* or *bin2-1* mutants and found that the BR-mediated down-regulation of *ACS* genes was greatly reduced in these two mutants compared with *det2-9* (S8 Fig), further indicating BZR1 or BES1 mediated BR signaling negatively regulates the expression *ACS* transcription factors. This result indicates that native physiological levels of BRs negatively regulate ethylene production through BZR1 or BES1 mediated transcriptional regulation of *ACS*s.

Since our results and Zhu et al.’s observations [49] are in contrast to other reports which showed that BRs enhanced ethylene biosynthesis [26, 46–48], we further did dosage-dependent assay to test the effects of BR on ethylene productions and root growth. Not surprisingly, root growth was inhibited gradually by eBL at concentrations ranging from 10 to 5000 nM (Fig 4A and 4B). However, ethylene content was greatly reduced in seedlings treated with low concentration of eBL (10 or 100 nM) while it was strongly increased when the concentration of eBL greater than 500 nM (Fig 4D). Root growth analysis under both treatment suggests that both high and low levels of ethylene cause a short-root phenotype (Fig 4D), which is consistent with the previous reports. In the octuple *acs* mutant (*CS16651*), which has only 10% ethylene level compared with WT, a reduced root growth phenotype was observed [53]. The *acs9* mutant also displays a short root phenotype (Fig 5E). The high levels of BR (500 nM or 1000 nM BR) induced ethylene production is also consistent with the previous reports in *Arabidopsis* [26, 46]. This study together with previous reports clearly showed that BRs either positively or negatively regulate ethylene biosynthesis in a concentration-dependent manner to control root growth. Certainly, since BR can also interact with other plant hormones such as auxin, ABA, cytokinin and jasmonic acid to regulate myriad aspects of plant growth and developmental processes in plants [54, 55], externally applied BR treatment caused root-growth phenotype might be also result from the interaction between BR and other plant hormones.

ROS represent not only a by-product of stress response, but also influence growth and development in response to both internal developmental signals and external environmental cues [28]. The contrasting ROS status in the cell proliferation and the cell differentiation zones has recently been shown to be an important driver of root growth [29]. A mitochondria localized P-loop NTPase was also reported to regulate quiescent center cell division and distal stem cell identity through the regulation of ROS homeostasis in *Arabidopsis* root [56]. It has been pointed out that ABA-promoted ROS regulates root meristem activity [31]. In cucumber plants exposed to exogenous BR, H_2_O_2_ accumulates as a result of an increased activity of NADPH oxidase [27], while in tomato, the same result is achieved by the up-regulation of *RBOH1* [57]. BR has been documented as inducing a receptor-dependent increase in cytosolic Ca^2+^, which stimulates NADPH oxidase-dependent ROS production [32, 58]. Thus, although the participation of BR in root growth and development is accepted, its interaction with ROS signaling has not been systematically explored to date. Here, a key finding was that the *det2-9* mutant hyper-accumulated O_2_^-^, which in itself likely contributed to the short root phenotype (Fig 7). BR inhibited the synthesis of O_2_^-^ via the peroxidase (Fig 8C and 8D and S11 Fig) rather than via the NADPH oxidase (Fig 8A and 8B and S10 Fig) pathway. These results suggest that H_2_O_2_ and superoxide anion respond dissimilarly to BR in *A*. *thaliana* seedlings. While the level of H_2_O_2_ rises rapidly upon exposure to exogenous BR, the one of the superoxide anion is repressed. In addition, the hyper-accumulation of ethylene displayed by *det2-9* contributed to a rise in the superoxide anion content in a peroxidase-independent manner (Fig 9A and 9B).

**Fig 10:**
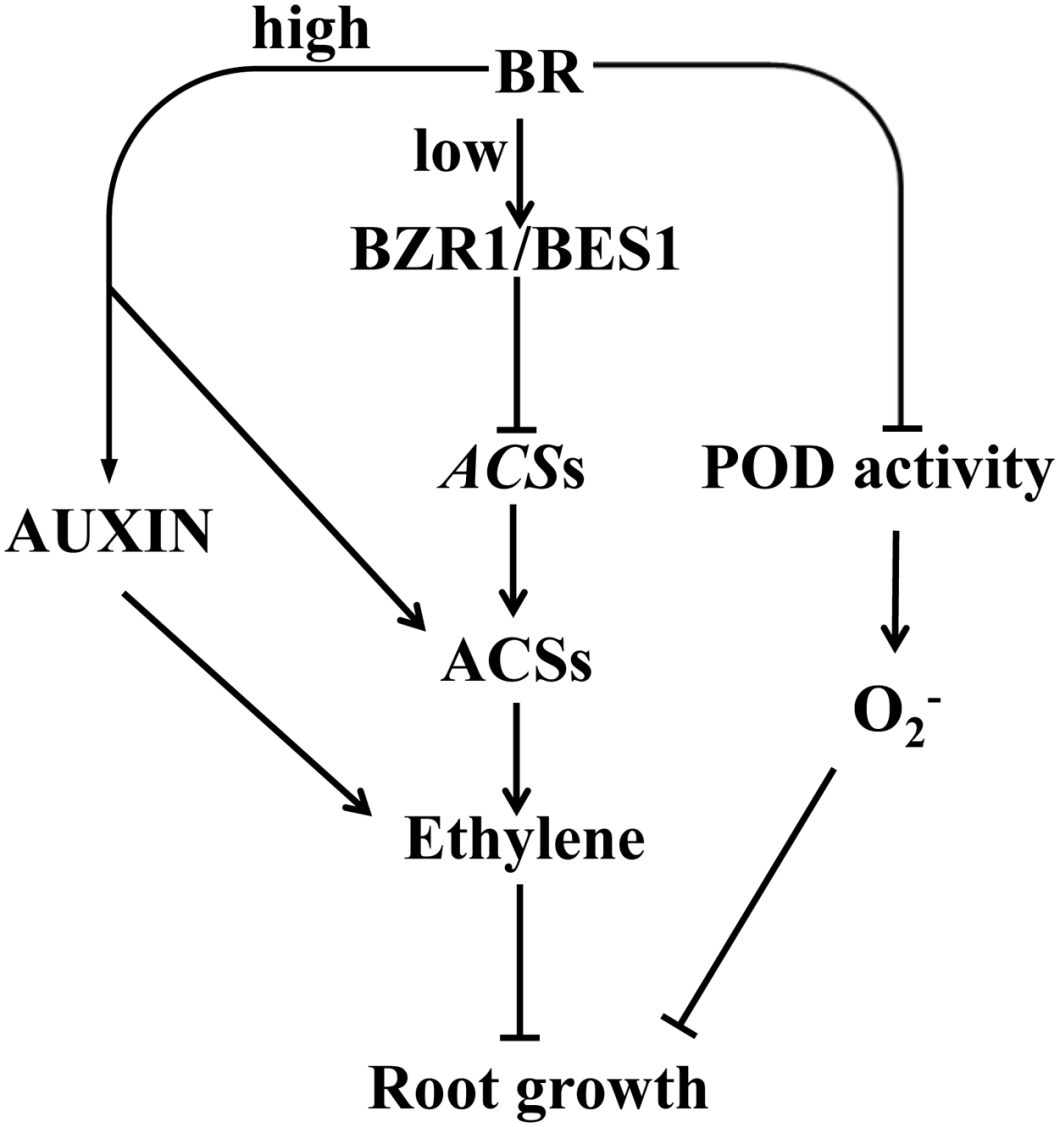
Proposed model to explain how BR regulates root growth in *A*. *thaliana*. BR inhibits ethylene synthesis by activating the transcription factors BZR1 and BES1 to repress the transcription of *ACS*s under low levels. While high levels of BR induce ethylene biosynthesis either through increasing the stability to ACSs or influencing auxin signaling regulated ethylene. At the same time, BR inhibits the synthesis of O_2_^-^ via the peroxidase pathway, but not NADPH oxidase pathway, which serves to regulate the growth of the *A*. *thaliana* seedling root.

In summary, according to this study together with the previous reports, a proposed model was given in Fig 10. We suggest that BR inhibits ethylene synthesis by activating the transcription factors BZR1 and BES1 under low levels. These transcription factors bind directly to the *ACS* promoters, thereby suppressing *ACS* expression and damping the level of ethylene synthesis under normal growth conditions. While high levels of BR induce ethylene biosynthesis either through increasing the stability of ACSs or influencing auxin signaling regulated ethylene production [47, 59, 60]. The possible regulation mechanism of BES1/BZR1’s activity under different levels of BR maybe refer to the regulation mechanism of ARF3 under different levels of auxin. Recent study has found that ARF3 acts as a repressor or activator depends on auxin concentration [61]. At the same time, BR inhibits the synthesis of O_2_^-^ via the peroxidase pathway, but not NADPH oxidase pathway, which serves to regulate the growth of the *A*. *thaliana* seedling root. The accumulation of the O_2_^-^ is also partially controlled by ethylene signaling in a peroxidase-independent manner and the O_2_^-^ accumulation can enhance ethylene signaling by increasing the expression of *ACS*s and *ACO*s. Understanding how ethylene mediates BR signaling to control the accumulation of the O_2_^-^ represents a logical follow-up research target.

## Materials and methods

### Plant materials and growing conditions

All of the *A*. *thaliana* mutants and/or transgenic lines utilized are in a Col-0 background; the following have been described elsewhere: *det2-1* [62], *bes1-D* [13], *bri1-116* [63], *bin2-1* [64], *p35S*::*BRI1-GFP* [65], *ein2-5* [66], *ein3/eil1-1* [67], *acs9* [68], *p35S*::*BZR1-YFP* [69], *pNP*::*BES1-FLAG* [70], *p35S*::*EIN3-GFP* [71], and *p35S*::*NADPH-GFP* [72]. And *rbohD*, *rbohF*, *rbohD/F* all described in Torres’ paper [73]. The octuple *acs* mutant (*CS16651*, *acs2-1/acs4-1/acs5-2/acs6-1/acs7-1/acs9-1/amiRacs8acs11*) [53] was obtained from the Arabidopsis Biological Resource Center (ABRC, Columbus, OH, USA), and the marker lines *pCYCB1;1*::*GUS* [74] and *pEBS*::*GUS* [75] from early research. The 1501-bp upstream region from the *DET2* start cordon and the cDNA of *DET2* were amplified and linked to the GFP-GUS reported in gateway vector PKGWFS7.1 [76] to obtain *pDET2*::*DET2-GFP-GUS* reporter construct. Prior to germination, the seed was surface-sterilized by fumigation in chlorine gas, held for two days at 4°C on solidified half strength Murashige and Skoog (MS) medium, then transferred to a growth room providing a 16 h photoperiod and a constant temperature of 20°C.

### Microscopy, growth measurement and histochemical GUS staining

Root tips were imaged by laser-scanning confocal microscopy. The number (obtained from a count of cells in the cortex file extending from the quiescent center to the TZ) and length of cortical and mature epidermal cells were obtained from microscope images using ImageJ software. The criteria for defining the MZ and TZ were those described by Napsucialy-Mendivil et al. [77]. The cell production rate was based on the rate of root growth and the length of fully elongated cells, and the cell cycle time on cell production and the number of cells present in the MZ, as described by Napsucialy-Mendivil et al. [77]. The number of cells displaced from the cell proliferation domain (N_transit_) during a 24 h period was estimated from the equation N_transit_ = (24 ln2 N_MZ_)/T, where N_MZ_ represents the number of cells in the RAM MZ and T means cell cycle time in hours. Histochemical GUS staining was performed according to the method described by Gonzalez-Garcia et al. [78].

### Map-based cloning of the gene underlying the *sr5* mutation

The mapping population was the F_2_ generation of the cross *sr5* x Landsberg erecta. Genomic DNA was extracted from each F_2_ seedlings showing the *sr5* phenotype. Simple sequence length polymorphism markers were used for the initial genome-wide linkage analysis, following Lukowitz et al. [79]. To enable fine mapping, 22 PCR-based markers were designed to target the relevant region of the *A*. *thaliana* genome sequence.

### RNA-Seq

RNA was isolated from the roots of six day-old *det2-9* and WT seedlings using the TRIzol reagent (Invitrogen, Carlsbad, CA, USA) and treated with DNase I to remove contaminating genomic DNA. The preparation was enriched for mRNA by introducing magnetic beads coated with oligo (dT). The resulting mRNA was fragmented into fragments of about 200 nt, and the cDNA first strand was then synthesized via random hexamer priming. After synthesizing the second strand with DNA polymerase I, the ds cDNA was purified using magnetic beads coated with oligo (dT) and End reparation is then performed. Adaptors were then ligated to each end of the fragments, and the products were size-selected by gel electrophoresis. Finally, the fragments were amplified based on the adaptor sequences, purified using magnetic beads coated with oligo (dT) and dissolved in the appropriate amount of Epstein-Barr solution. The concentration and integrity of the ds cDNA was monitored using a 2100 Bioanaylzer device (Agilent Technologies Japan Ltd.). The cDNA was then sequenced using an Ion Proton platform (www.thermofisher.com). Low quality and adaptor sequences were removed and the remaining sequences were then aligned to the *A*. *thaliana* genome sequence using SOAP2 software. Individual transcript abundances were expressed in the form of the number of reads per kilobase per million reads (RPKM), and differentially transcribed genes were identified using the thresholds FDR≤0.001 and |log_2_|≥1 [80].

### qRT-PCR

The RNA template required for qRT-PCR was isolated using an RNeasy PlantMini kit (Qiagen, Hilden, Germany) following the manufacturer’s protocol. After treating with DNase I to remove contaminating genomic DNA, a 2 μg aliquot was reverse-transcribed using a Transcriptor First Strand cDNA Synthesis kit (Roche, Basel, Switzerland), following the manufacturer’s protocol. The subsequent qRT-PCRs were run on a MyiQTM Real-time PCR Detection System (Bio-Rad, Hercules, CA, USA) using FastStart Universal SYBR Green Master mix (Roche, Basel, Switzerland). Each sample was represented by three biological replicates, and each biological replicate by three technical replicates. The reference sequence was *AtACTIN2* (*At3g18780*). Primer sequences are given in S2 Dataset.

### Ethylene quantification

Ten seedlings were placed in a 100 mL vial containing 50 mL solidified half strength MS either with or without eBL or PPZ, and immediately capped. The vials were held under a 16 h photoperiod and a constant temperature of 20°C. After seven days, a 10 μL sample of the headspace was subjected to gas chromatography using a GC-6850 device equipped with a flame ionization detector (Agilent Technologies Japan Ltd.).

### Yeast one-hybrid assay

The coding sequences of *BES1* and *BZR1* were inserted separately into the *Eco*RI-*Xho*I cloning site of pGADT7 (Takara, USA), while the promoter sequences of *ACS6*, *7*, *9*, *11*, *ACO1* and *3* were inserted into the cloning site of pAbAi. The primer sequences used in the construction of the various constructs are given in S2 Dataset. Each of the constructs (including an empty vector for control purposes) was transferred separately into yeast Y1HGold using the PEG/LiAc method. The yeast cells were plated onto SD/-Ura/-Leu medium containing various concentrations of Aureobosidin A to allow for a highly stringent screening of interactions. The procedure followed the manufacturer’s protocol given for the Matchmaker Gold Yeast One-Hybrid Library Screening System (www.clontech.com).

### Chromatin immunoprecipitation (ChIP)

Ten day old transgenic plants were used for the ChIP assay following Gendrel et al. [81]. The quantity of precipitated DNA and input DNA was detected by qPCR. For each *ACS* promoter, primers were designed to amplify a fragment of length ~70–150 bp lying within the 2 kbp of sequence upstream of the transcription start site. The relevant primers are given in S2 Dataset. Enrichment was calculated from the ratio of bound sequence to input.

### Transient expression

The *BES1* or *BZR1* coding sequences were amplified and the resulting sequences introduced into pBI221 to place them under the control of the CaMV 35S promoter. The *ACS* promoter sequences were amplified and introduced into the pGreenII0800-LUC reporter vector. Both recombinant plasmids were then transferred into *A*. *thaliana* protoplasts. Firefly luciferase (LUC) and renillia luciferase (REN) activities were measured using the Dual-Luciferase Reporter Assay System (www.promega.com). LUC activity was normalized against REN activity [82]. Details of all primers used are given in S2 Dataset.

### NBT assay for the superoxide anion

The roots of five day-old seedlings were immersed for 15 min in 2 mM NBT in 20 mM phosphate buffer (pH 6.1). The reaction was stopped by transferring the seedlings into distilled water. The material was then imaged under a light stereomicroscope.

### Peroxidase activity measurement

Tissue peroxidase activity was measured by a spectrophotometric analysis (420 nm) of the formation of purpurogallin from pyrogallol in the presence of H_2_O_2_. The roots of nine day-old seedlings were harvest and weighted. Tissue homogenate was prepared using 9 times phosphate buffer and then centrifuged for 10 min in 3500 rpm. The supernatant was used for peroxidase activity measurement. A single unit of enzyme was defined as the amount catalyzed and generated 1 μg pyrogallol by 1.0 mg fresh tissues in the reaction system at 37°C. Peroxidase activity was calculated from the formula provided with the peroxidase assay kit (Jiancheng Bioengineering Institute, Nanjing, China).

### Accession numbers

Sequence data for genes used in this study can be found in the Arabidopsis Genome Initiative or GenBank/EMBL databases under the following accession numbers:

*DET2* (*At2g38050*), *BES1* (*At1g19350*), *BZR1* (*At1g75080*), *ACS1* (*At3g61510*), *ACS2* (*At1g01480*), *ACS4* (*At2g22810*), *ACS5* (*At5g65800*), *ACS6* (*At4g11280*), *ACS7* (*At4g26200*), *ACS9* (*At3g49700*), *ACS11* (*At4g08040*), *WOX5* (*At3g11260*), *ARF10* (*At2g28350*), *ARF16* (*At4g30080*), *ARR1* (*At3g16857*), *SHY2* (*At1g04240*), *BRI1* (*At4g39400*), *CYCB1;1* (*At4g37490*), *ACO1* (*At2g19590*), *ACO2* (*At1g62380*), *ACO3* (*At2g05710*), *ACO4* (*At1g05010*), *ACO5* (*At1g77330*), *ERF6* (*At4g17490*), *ERF13* (*At2g44840*), *ERF17* (*At1g19210*), *ERF104* (*At5g61600*), *ERF105* (*At5g51190*), *EBS* (*At4g22140*), *EIN3* (*At3g20770*), *EIL1* (*At2g27050*), *RBOHC* (*At5g51060*), *RBOHD* (*At5g47910*), *RBOHF* (*At1g64060*), *RBOHG* (*At4g25090*), *TCH4* (*At4g57560*), *BAS1* (*At2g26710*), *IAA17* (*At1g04250*), *IAA19* (*At3g15540*), *ACTIN2* (*At3g18780*).

## Supporting information

S1 Fig Positional cloning of the gene underlying the *sr5* mutation. (A) The mutated gene maps to chromosome 2. The *sr5* allele sequence differs from the WT allele of *At2g38050* by a point mutation causing a shift from G to A at position 107. (B) Phenotype of five day-old *sr5* and *det2-1* seedlings exposed to eBL (10 nM) either under lit or non-lit conditions. Bar = 1 cm. (C) Root phenotype of five day-old seedlings of the F_1_ hybrid *sr5 x det2-1* and its reciprocal. Bar = 1 cm. (D) Root phenotype of a five day-old *sr5* seedling carrying the transgene *pDET2*::*DET2-GFP-GUS*. Bar = 1 cm. (E) Phenotype of WT, *sr5* and *det2-1* 17 day-old seedlings. Bar = 1 cm.
(TIF)

S2 Fig GUS expression in five-day old *sr5* seedling carrying the transgene *pDET2*::*DET2-GFP-GUS*. Bar = 50 μm.
(TIF)

S3 Fig Relative transcript abundance of BR induced genes in seedlings of WT, *det2-9* and *det2-1*. (TIF)

S4 Fig Relative transcript abundance of ACC synthase genes (*ACS2*, *4*, *6*, *7*, 8, *9*, *11*) in dark-grown seedlings of WT and *det2-9*. **: means significant difference compared to control (*P*<0.01).
(TIF)

S5 Fig The *det2-9* mutant accumulates more ethylene than WT when grown in darkness. Ethylene production by five day-old seedlings of various BR-related transgenic and WT seedlings exposed to either eBL(10 nM) or propiconazole (2 μM) in dark conditions. Data shown are mean±SE (n = 5). **: means significant difference compared to control (*P*<0.01).
(TIF)

S6 Fig Effect of ethylene on cell length and cell number in RAM. (A) Cortical cell length in the maturation zone of five day-old WT and *det2-9* seedlings when treated with AgNO_3_ or ACC. Data shown are mean±SE (n = 25), Different letters associated with values indicate a significant difference (*P*<0.01). (B) Cell number in the proliferation domain of five day-old WT and *det2-9* seedlings when treated with AgNO_3_ or ACC. Data shown are mean±SE (n = 25), Different letters associated with values indicate a significant difference (*P*<0.01).
(TIF)

S7 Fig Neither BES1 nor BZR1 interact directly with the *ACO1* or *ACO3* promoters, as indicated by a yeast one-hybrid binding assay. (TIF)

S8 Fig A qRT-PCR analysis of genes involved in ethylene production in BR mutants. Relative transcript abundance of ACC synthase genes (*ACS6*, *7*, *9*, *11*) in WT, *det2-9*, *bri1-116* and *bin2-1* when treated with or without eBL (10 nM).
(TIF)

S9 Fig The production of H_2_O_2_ in the *det2-9* mutant and WT five-day-old seedlings. WT and *det2-9* roots are stained by DAB to quantify H_2_O_2_ levels. Bar = 50 μm.
(TIF)

S10 Fig The BR-mediated inhibition of superoxide anion synthesis does not operate through the NADPH oxidase pathway. (A) Transcription of *RBOH* genes, assayed by qRT-PCR in WT and *det2-9* seedlings. (B) Relative root length in the mutants *rbohD*, *rbohF* and *rbohD/F* in the presence or absence of propiconazole (2 μM). Data shown are mean±SE (n = 30). (C) NBT staining of root of WT, *35S*::*NADPHD-GFP* and *rbohD/F* plants exposed to propiconazole (2 μM). Bar = 50 μm. (D) NBT staining of root of WT, *p35S*::*EIN3-GFP* and *ein3/eil1-1* plants exposed to eBL (10 nM) or propiconazole (2 μM). Bar = 50 μm. (E) NBT staining of root of WT, *det2-9*, *rbohD*, *rbohD/F*, *det2-9/rbohD* and *det2-9/rbohD/F* plants. Bar = 50 μm.
(TIF)

S11 Fig Primary root length of WT and *det2-9* seedlings when exposed to inhibitors of peroxidase. (TIF)

S12 Fig Transcription of genes encoding peroxidase in *det2-9* and WT, assayed by qRT-PCR. (TIF)

S13 Fig NBT staining of root of WT, *p35S*::*EIN3-GFP* and *ein3/eil1-1* plants exposed to eBL(10 nM) or propiconazole (2 μM). Bar = 50 μm.
(TIF)

S14 Fig Transcription of genes encoding ACC synthase (ACS) and ACC oxidase (ACO) when treated or not with MV, assayed by qRT-PCR. **: means in treated seedling significantly differ from untreated samples (*P*<0.01).
(TIF)

S1 Dataset Different expression genes related to ethylene in *det2-9*. (XLSX)

S2 Dataset List of primer sequences used in this paper. (XLSX)
