# Supplementary figures and images for: Brassinosteroids regulate root growth by controlling reactive oxygen species homeostasis and dual effect on ethylene synthesis in Arabidopsis
Source: PLoS Genet. 2018 Jan 11;14(1):e1007144. doi: 10.1371/journal.pgen.1007144 (PMC5783399; doi:10.1371/journal.pgen.1007144)

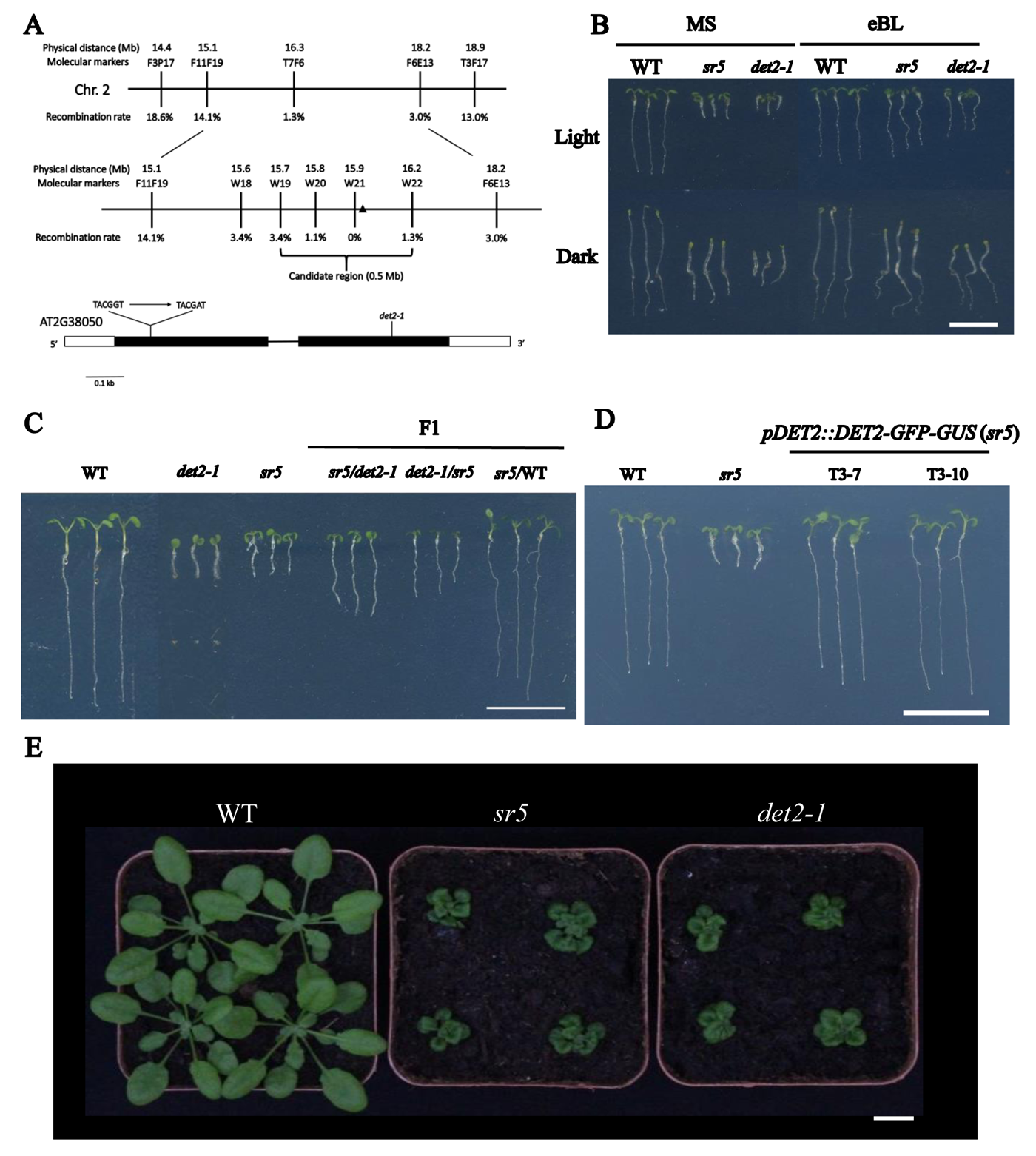

Supplement: S1 Fig — (A) The mutated gene maps to chromosome 2. The sr5 allele sequence differs from the WT allele of At2g38050 by a point mutation causing a shift from G to A at position 107. (B) Phenotype of five day-old sr5 and det2-1 seedlings exposed to eBL (10 nM) either under lit or non-lit conditions. Bar = 1 cm. (C) Root phenotype of five day-old seedlings of the F1 hybrid sr5 x det2-1 and its reciprocal. Bar = 1 cm. (D) Root phenotype of a five day-old sr5 seedling carrying the transgene pDET2::DET2-GFP-GUS. Bar = 1 cm. (E) Phenotype of WT, sr5 and det2-1 17 day-old seedlings. Bar = 1 cm. (TIF) [file pgen.1007144.s001.tif]

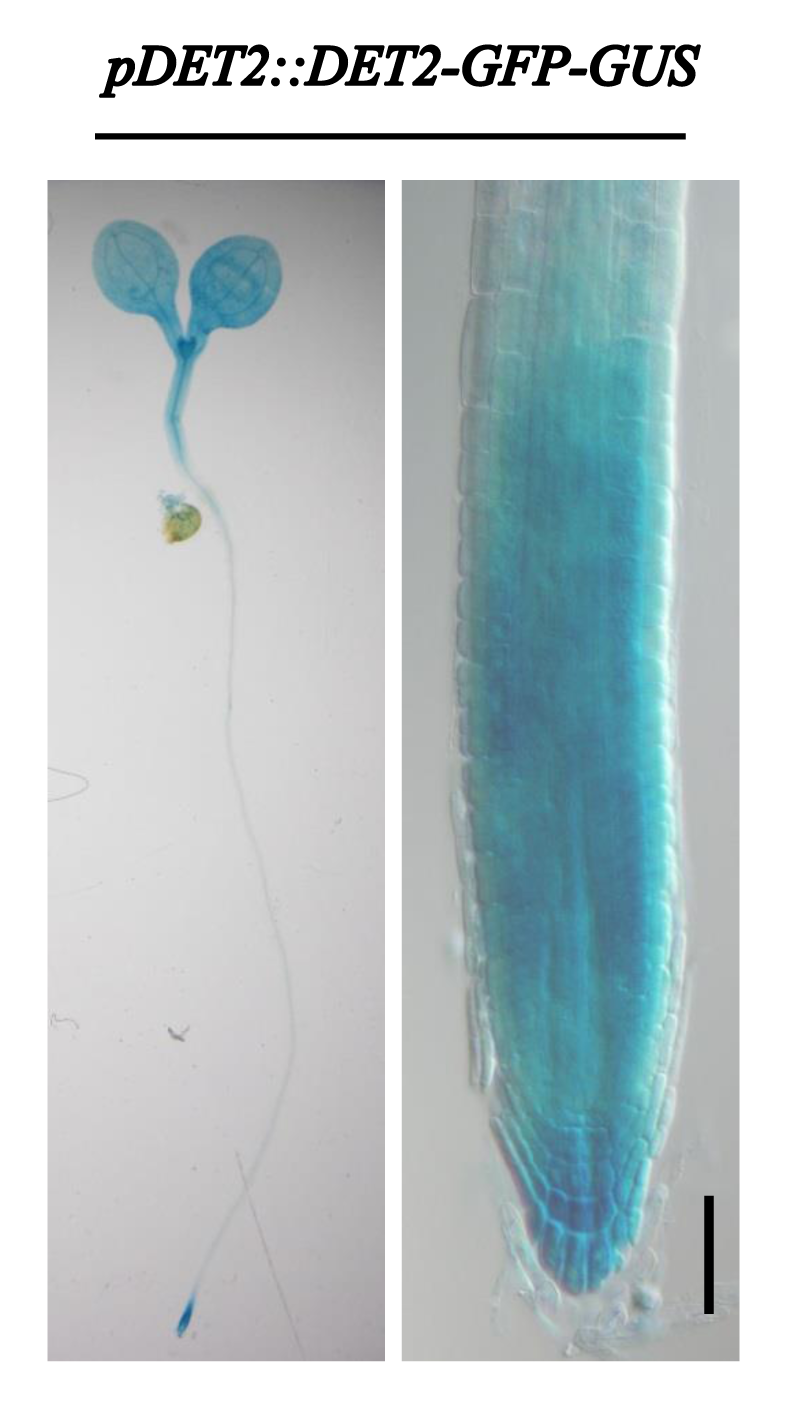

Supplement: S2 Fig — Bar = 50 μm. (TIF) [file pgen.1007144.s002.tif]

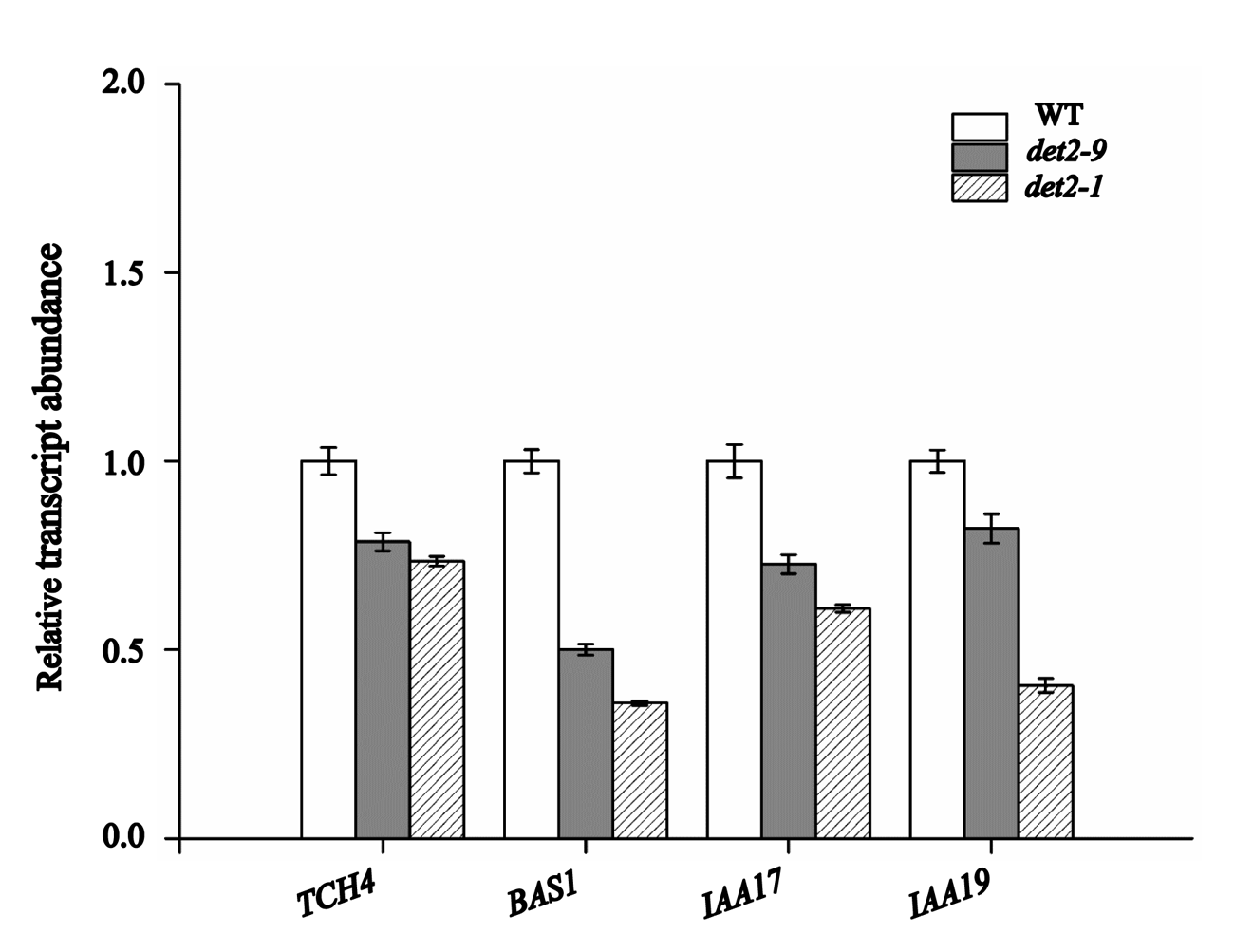

Supplement: S3 Fig — (TIF) [file pgen.1007144.s003.tif]

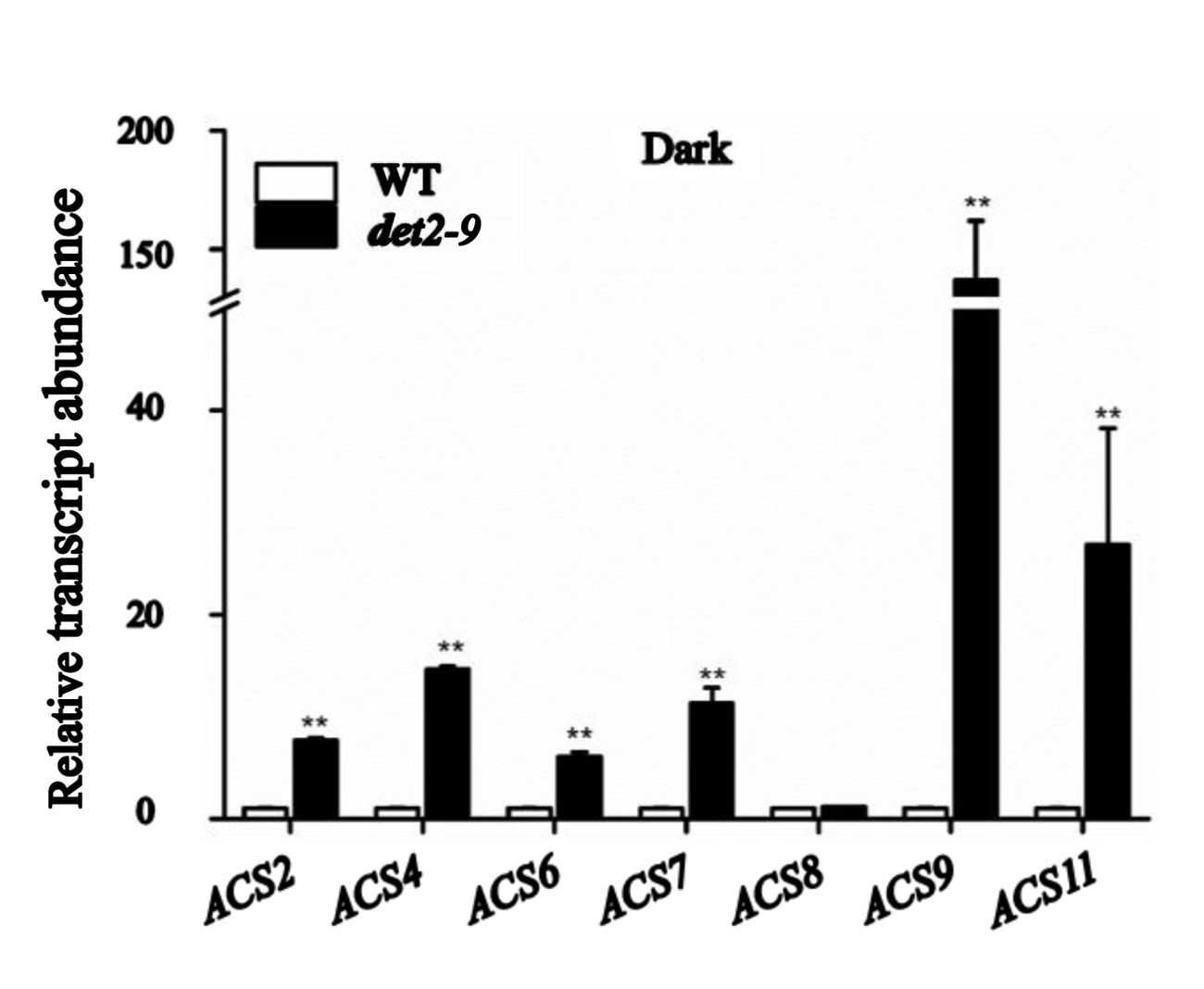

Supplement: S4 Fig — **: means significant difference compared to control (P<0.01). (TIF) [file pgen.1007144.s004.tif]

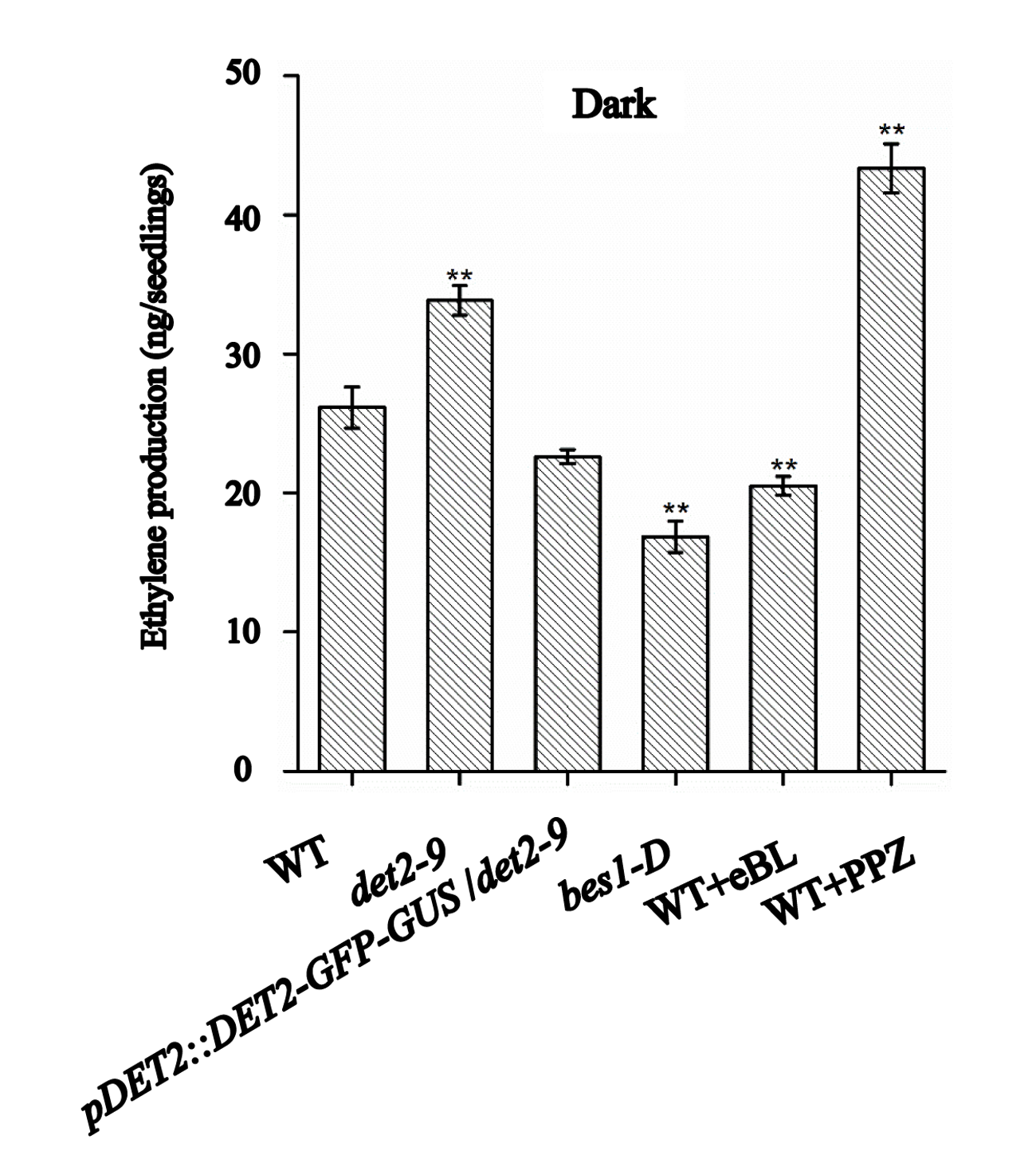

Supplement: S5 Fig — Ethylene production by five day-old seedlings of various BR-related transgenic and WT seedlings exposed to either eBL(10 nM) or propiconazole (2 μM) in dark conditions. Data shown are mean±SE (n = 5). **: means significant difference compared to control (P<0.01). (TIF) [file pgen.1007144.s005.tif]

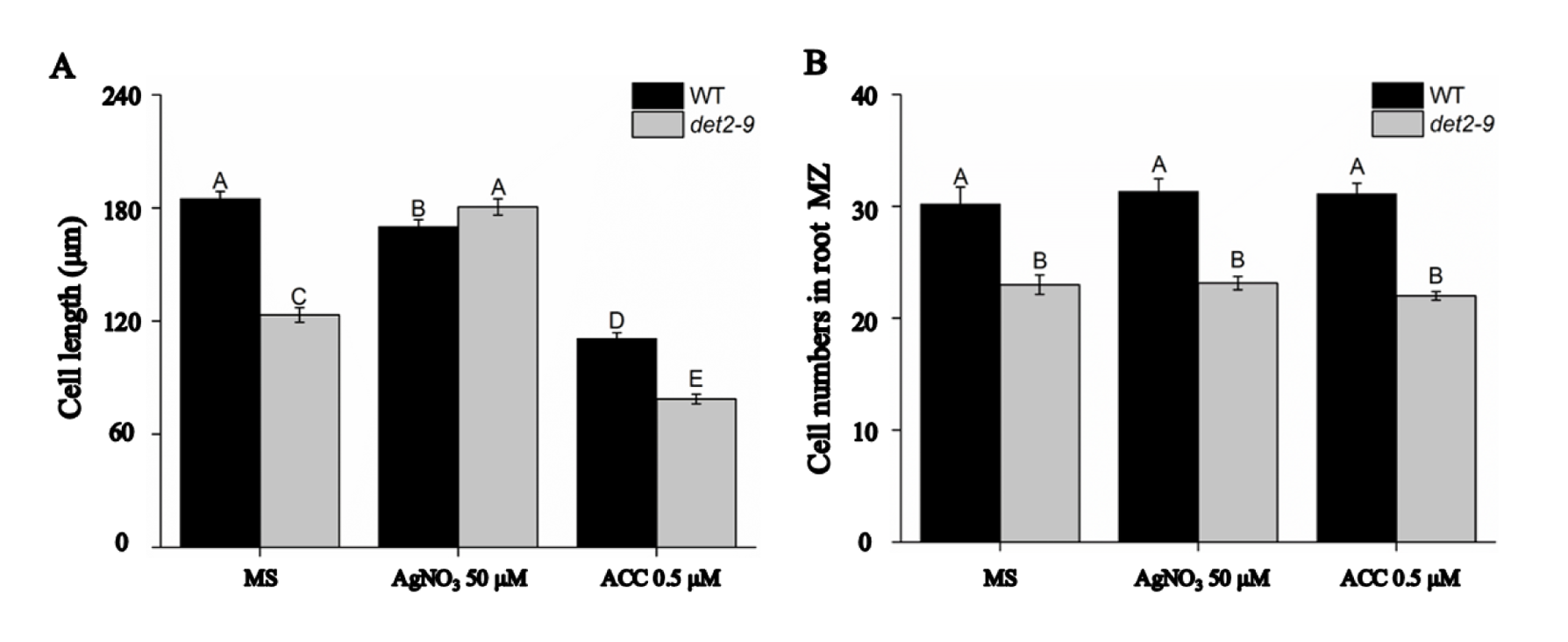

Supplement: S6 Fig — (A) Cortical cell length in the maturation zone of five day-old WT and det2-9 seedlings when treated with AgNO3 or ACC. Data shown are mean±SE (n = 25), Different letters associated with values indicate a significant difference (P<0.01). (B) Cell number in the proliferation domain of five day-old WT and det2-9 seedlings when treated with AgNO3 or ACC. Data shown are mean±SE (n = 25), Different letters associated with values indicate a significant difference (P<0.01). (TIF) [file pgen.1007144.s006.tif]

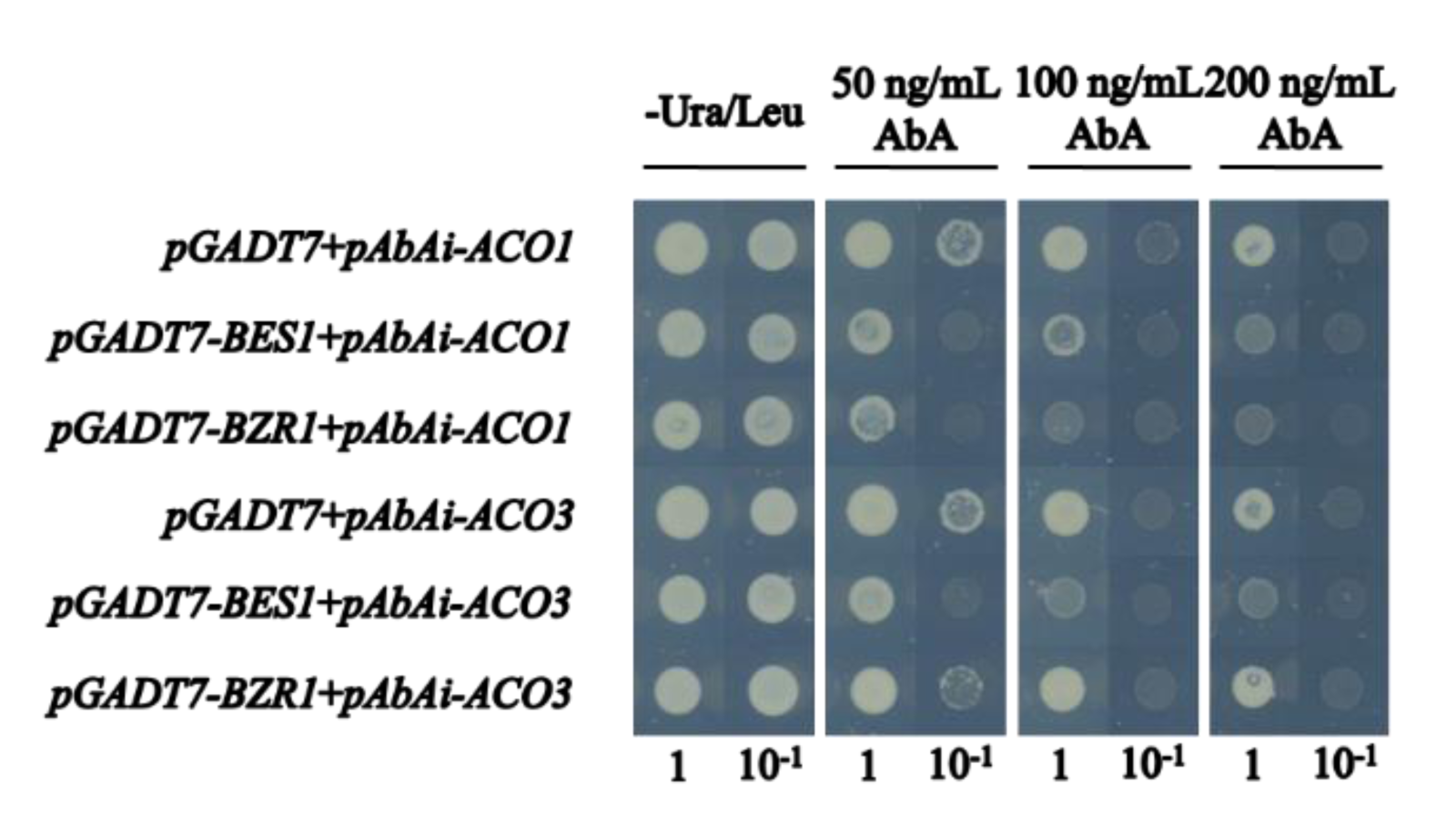

Supplement: S7 Fig — (TIF) [file pgen.1007144.s007.tif]

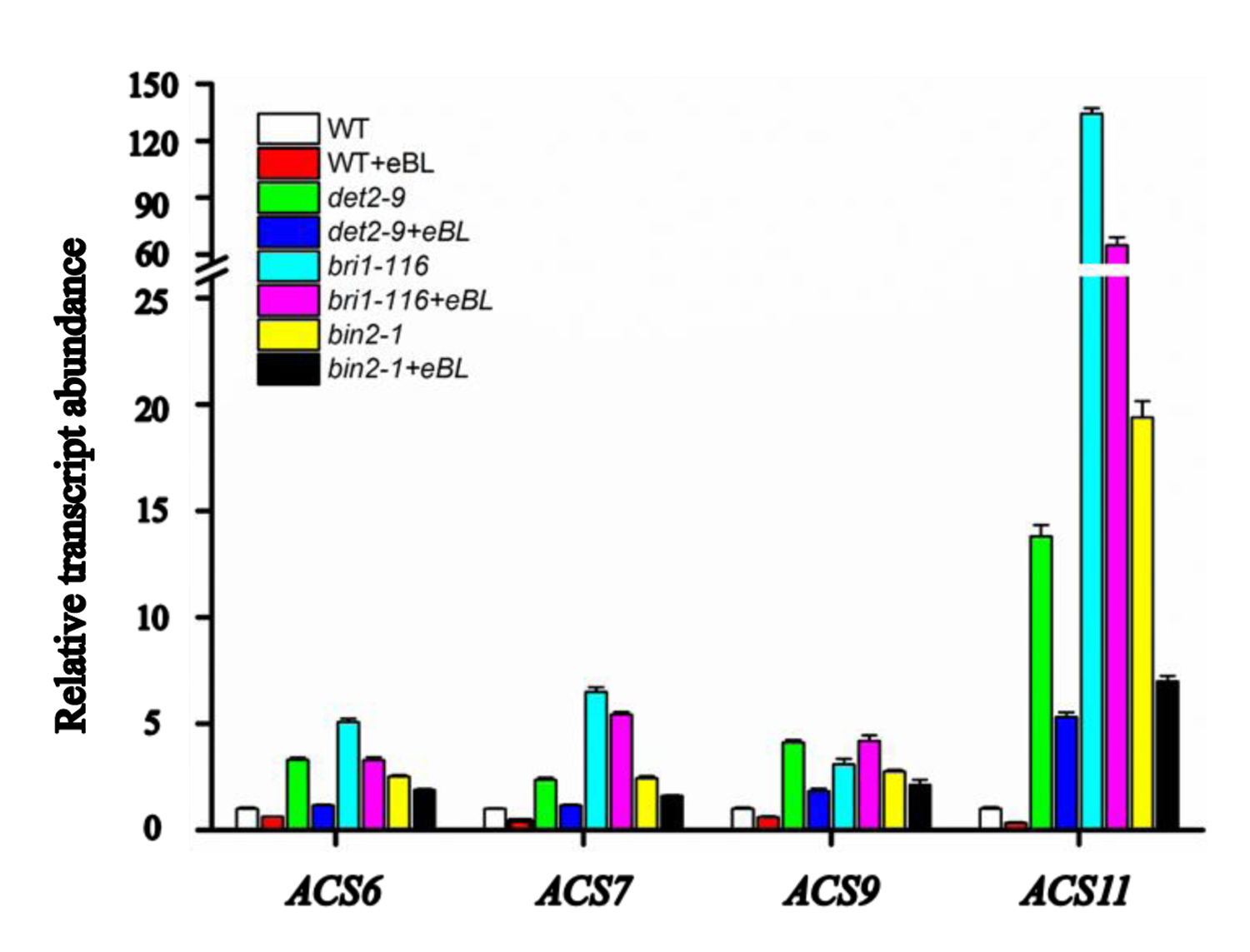

Supplement: S8 Fig — Relative transcript abundance of ACC synthase genes (ACS6, 7, 9, 11) in WT, det2-9, bri1-116 and bin2-1 when treated with or without eBL (10 nM). (TIF) [file pgen.1007144.s008.tif]

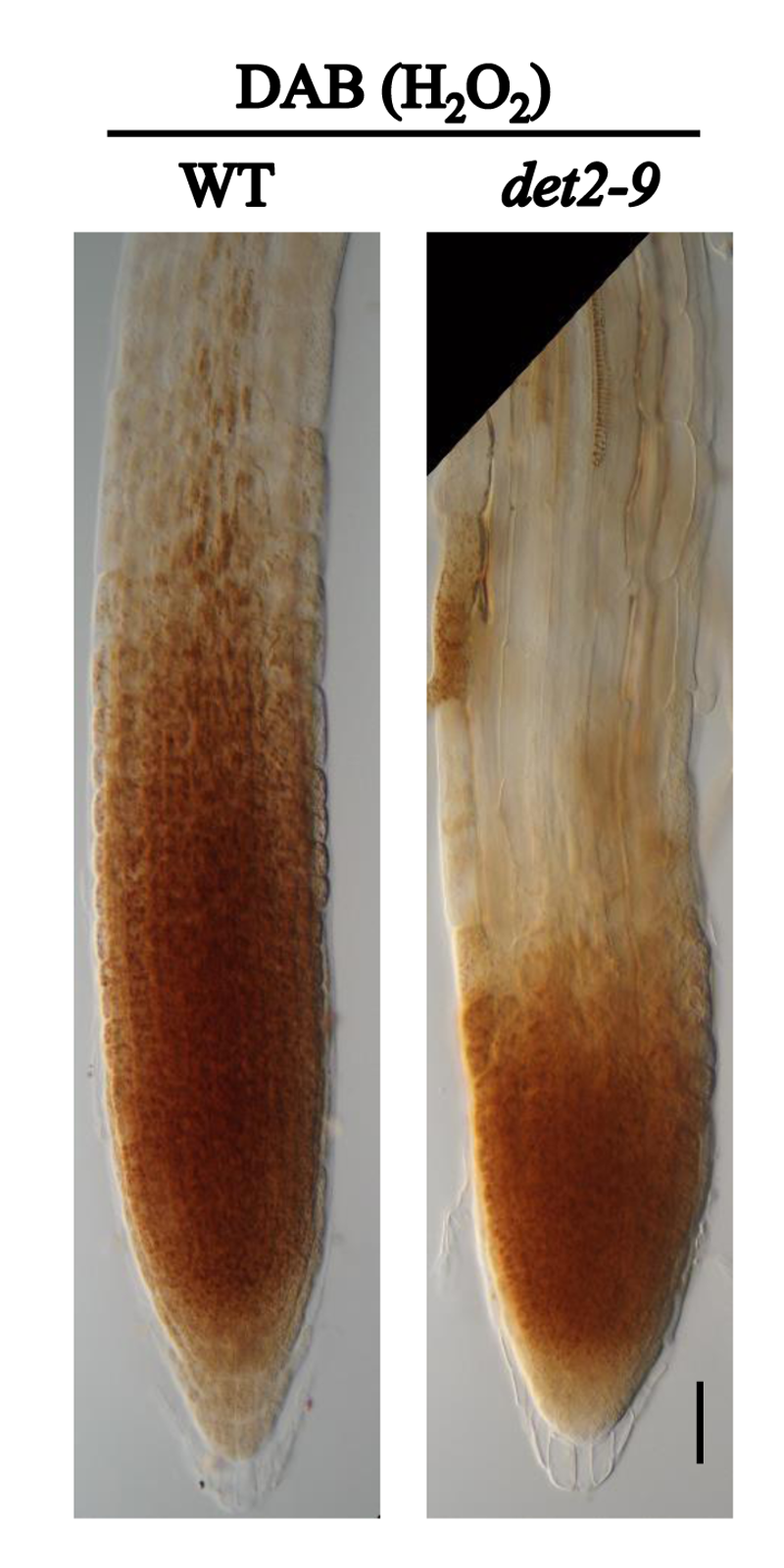

Supplement: S9 Fig — WT and det2-9 roots are stained by DAB to quantify H2O2 levels. Bar = 50 μm. (TIF) [file pgen.1007144.s009.tif]

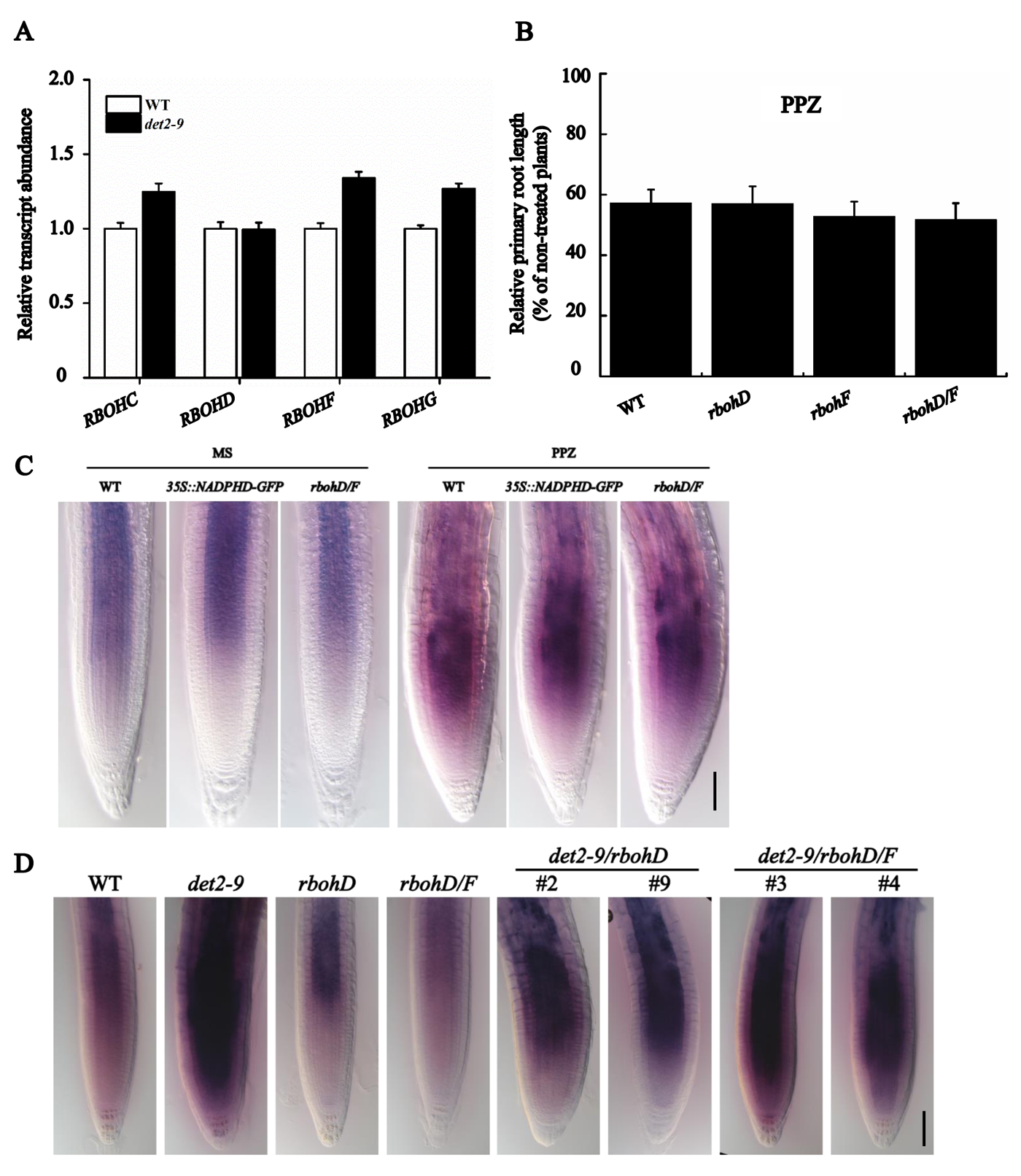

Supplement: S10 Fig — (A) Transcription of RBOH genes, assayed by qRT-PCR in WT and det2-9 seedlings. (B) Relative root length in the mutants rbohD, rbohF and rbohD/F in the presence or absence of propiconazole (2 μM). Data shown are mean±SE (n = 30). (C) NBT staining of root of WT, 35S::NADPHD-GFP and rbohD/F plants exposed to propiconazole (2 μM). Bar = 50 μm. (D) NBT staining of root of WT, p35S::EIN3-GFP and ein3/eil1-1 plants exposed to eBL (10 nM) or propiconazole (2 μM). Bar = 50 μm. (E) NBT staining of root of WT, det2-9, rbohD, rbohD/F, det2-9/rbohD and det2-9/rbohD/F plants. Bar = 50 μm. (TIF) [file pgen.1007144.s010.tif]

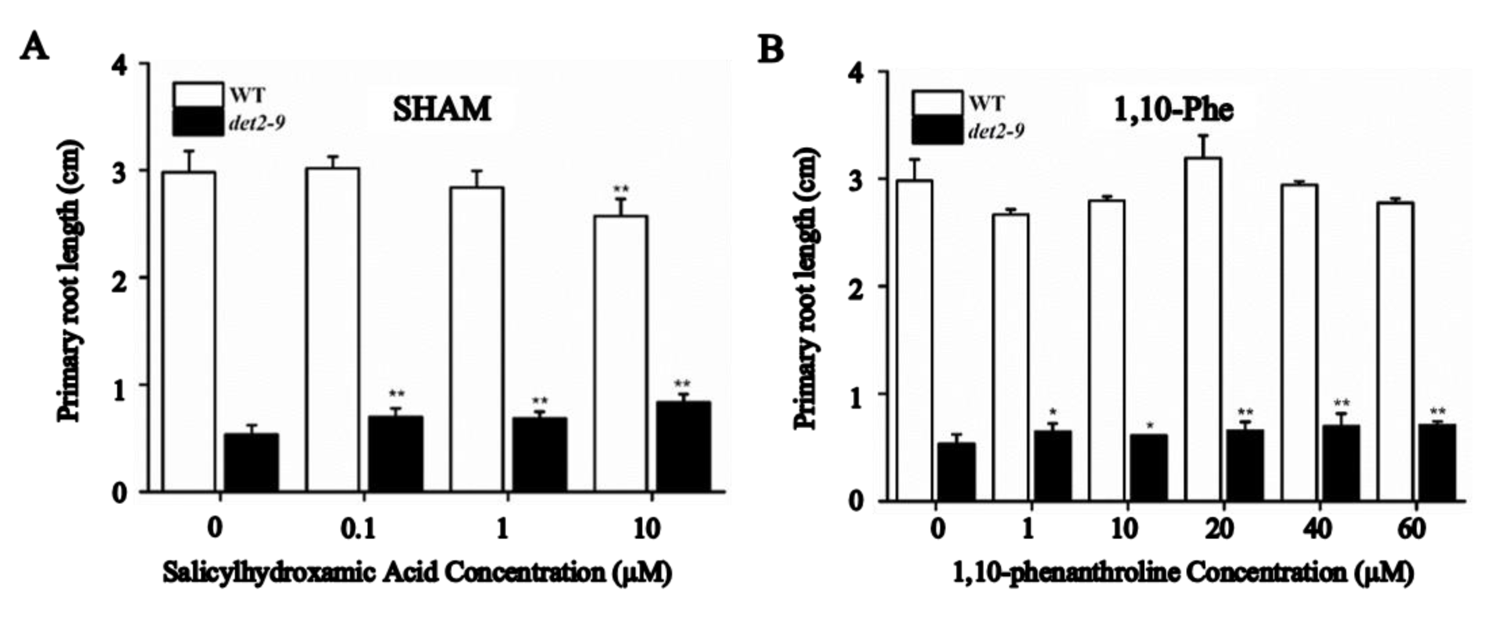

Supplement: S11 Fig — (TIF) [file pgen.1007144.s011.tif]

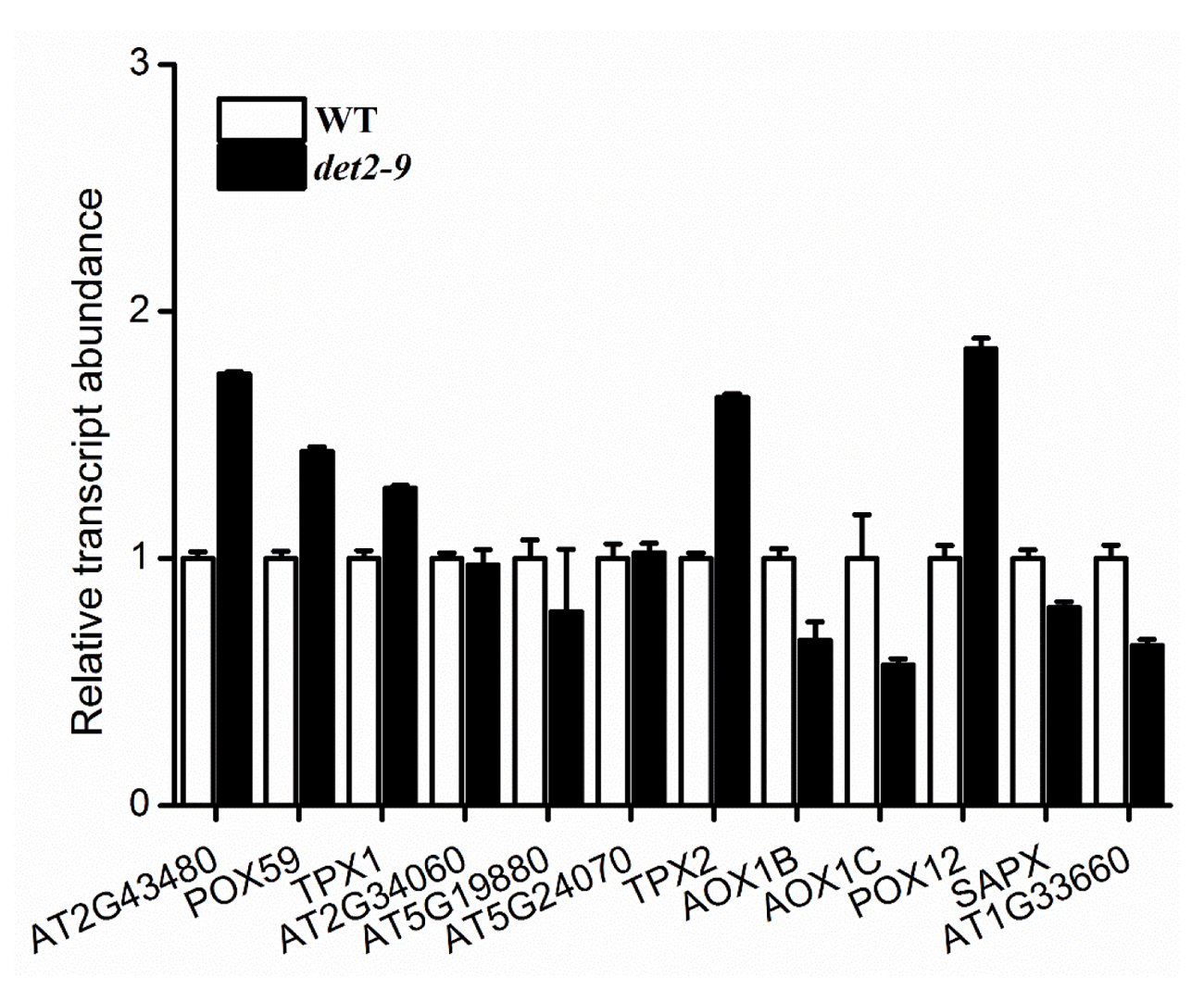

Supplement: S12 Fig — (TIF) [file pgen.1007144.s012.tif]

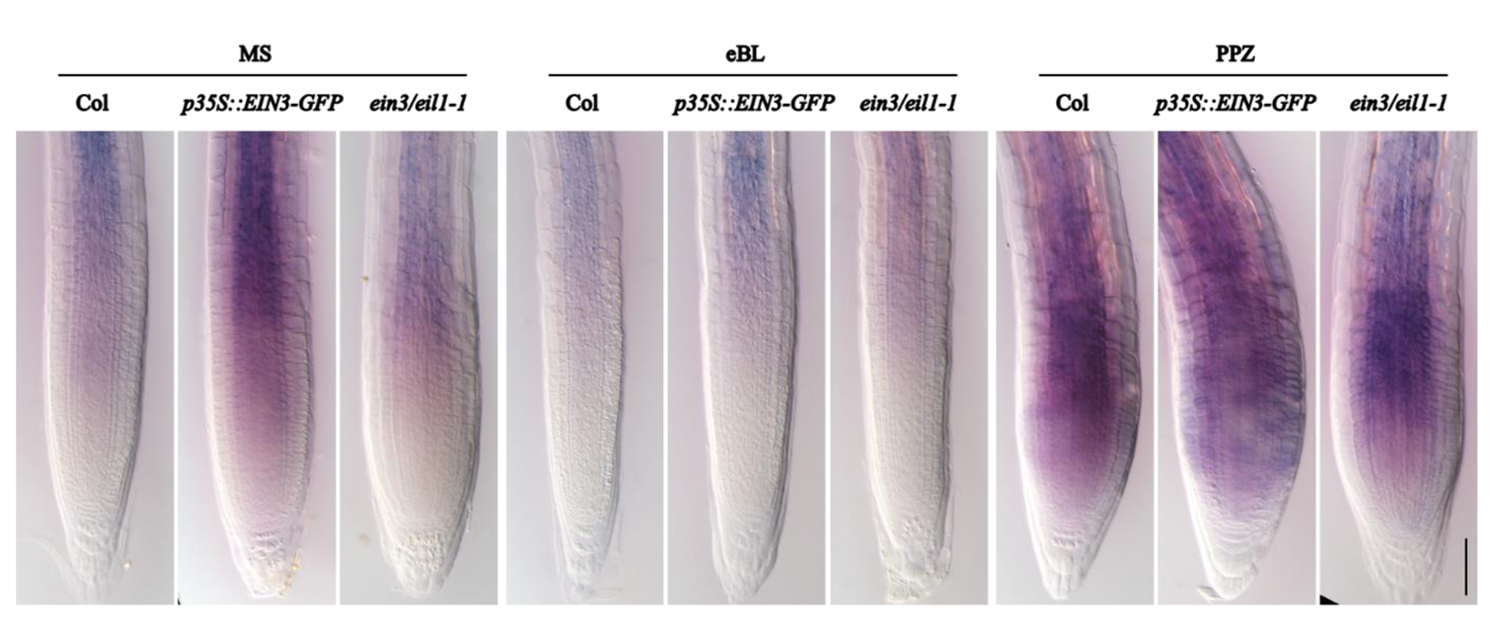

Supplement: S13 Fig — Bar = 50 μm. (TIF) [file pgen.1007144.s013.tif]

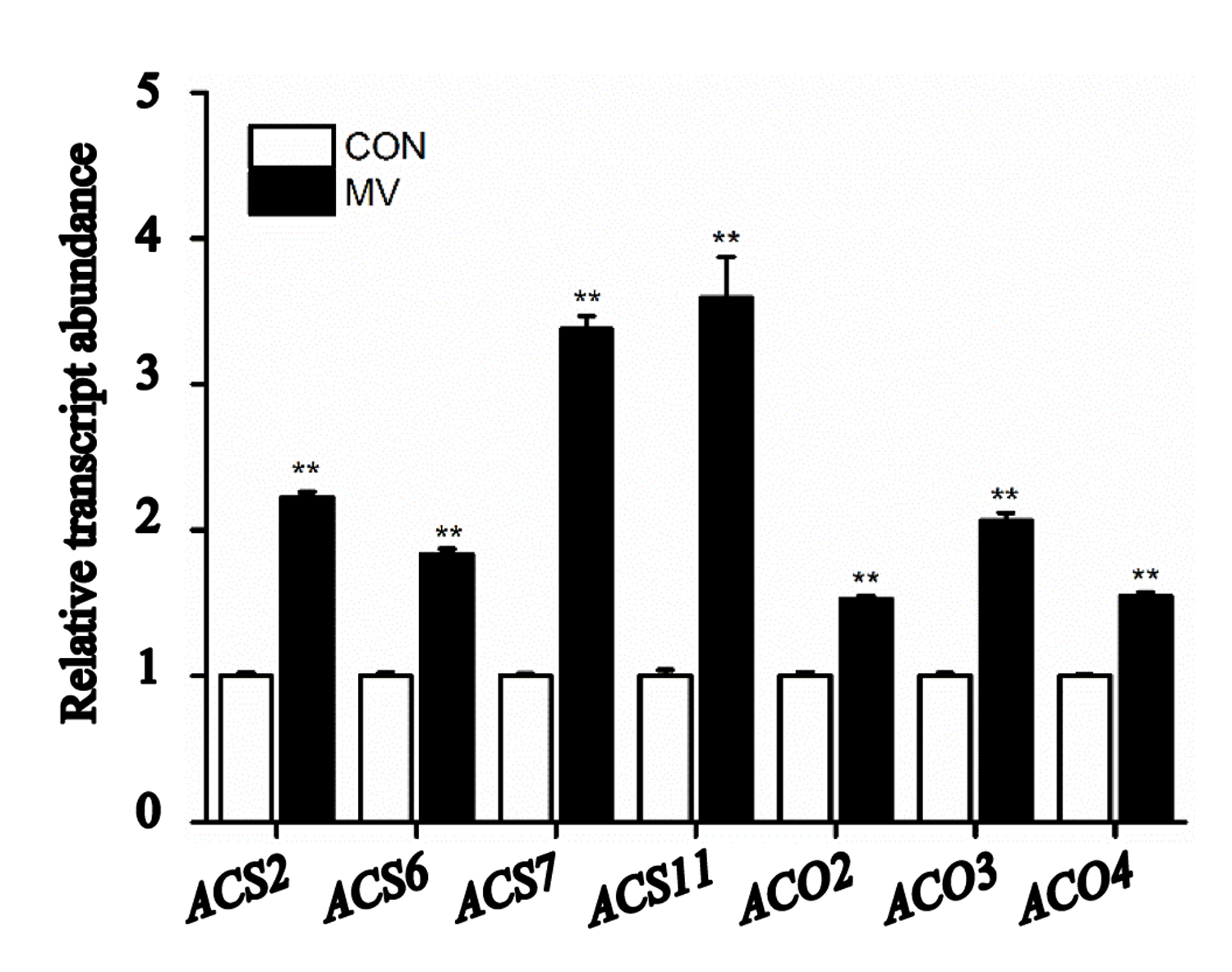

Supplement: S14 Fig — **: means in treated seedling significantly differ from untreated samples (P<0.01). (TIF) [file pgen.1007144.s014.tif]
